# Data on atmospheric ^129^I concentrations and ^129^I/^137^Cs ratios for suspended air particulate matter dispersed in eastern Japan just after the 2011 nuclear accident in Fukushima, Japan

**DOI:** 10.1016/j.dib.2022.108621

**Published:** 2022-09-28

**Authors:** Mitsuru Ebihara, Naoki Shirai, Yasuji Oura, Haruo Tsuruta, Hiroyuki Matsuzaki, Yuichi Moriguchi

**Affiliations:** a Department of Earth Sciences, Waseda University, 1-6-1 Nishi-waseda, Shinjuku-ku, Tokyo 169-8050, Japan; b Department of Chemistry, Tokyo Metropolitan University, 1-1 Minami-osawa, Hachioji, Tokyo 192-0397, Japan; c Remoto Sensing Technology Center of Japan, 3-17-1 Toranomon, Minato-ku 105-0001, Japan; d Department of Nuclear Engeneering and Management, School of Engineering, University of Tokyo, 2-11-16 Yayoi, Bunkyo-ku, Tokyo 113-0032, Japan; e National Institute for Environmental Studies, 16-2 Onogawa, Tsukuba, Ibaraki 305-8508, Japan

**Keywords:** Fukushima nuclear accident, Radioactive nuclides, ^129^I, ^137^Cs, Suspended particulate matter (SPM), Time-series variation, Metropolitan area

## Abstract

Data of the atmospheric activity concentrations (in Bq/m^3^) of ^129^I dispersed into the environment as aerosol immediately after the nuclear accident at Fukushima Daiichi Nuclear Power Plant in 2011 are presented. The radioactivity of ^129^I was determined in suspended particulate matter (SPM) collected on filter tapes at 41 SPM monitoring sites in Fukushima and other prefectures in eastern Japan including the metropolitan area. For quantitative determination of ^129^I in SPM samples by accelerator mass spectrometry (AMS), ^129^I was chemically separated. Prior to the ^129^I measurement, the ^137^Cs activity was determined for the same SPM sample by gamma-ray spectrometry using Ge-semiconductor detectors. Combining activity concentrations of the two nuclides, an activity ratio of ^129^I/^137^Cs (in Bq/Bq) was calculated for each SPM sample. In our research project, atmospheric activity concentrations of ^129^I and ^137^Cs, and their activity ratios were obtained for 920 SPM samples. Scientific discussion related to those data was described in the research article entitled “Time-series variations of atmospheric ^129^I concentrations and ^129^I/^137^Cs ratios in eastern Japan just after the 2011 nuclear accident in Fukushima, Japan” (Ebihara et al. 2022), where 363 data sets were presented. The remaining 557 data sets are presented in this article, so this data article makes up for the original research article (Ebihara et al. 2022). Blank values were obtained for whole analytical procedure. In addition, those for reagents and filters (both bland-new and used filters) were analyzed for assessing the contribution of the ^129^I activity from these samples. Those data also are presented in this article.

**Specifications Table**

**Table:** 

Subject	Environmental Science
Specific subject area	Pollution of radioactive aerosol containing fissiogenic ^129^I and ^137^Cs dispersed into the environment by the 2011 Fukushima nuclear power plant accident
Type of data	Table
How data were acquired	Data were obtained by the following methods; (i) Accelerator mass spectrometry (AMS) for the ^129^I radioactivity measurement, and (ii) Gamma-ray spectrometry for the ^137^Cs radioactivity measurement.
Data format	Raw and analyzed
Description of data collection	Suspended particulate matter (SPM) samples analyzed in this study have been hourly collected in Fukushima and other prefectures in eastern Japan during and after the 2011 Fukushima nuclear disaster. The radioactivity of ^129^I was determined by AMS using a tandem accelerator at the University of Tokyo after chemically separating ^129^I in SPM. The radioactivity of ^137^Cs was non-destructively determined by gamma-ray spectrometry at Tokyo Metropolitan University.
Data source location	For their ^129^I and ^137^Cs content measurements, used were the SPM samples which were collected at SPM monitoring stations located in Miyagi, Fukushima, Ibaraki, Saitama, Tokyo, Chiba and Kanagawa prefectures in eastern Japan. Data were obtained at the University of Tokyo and Tokyo Metropolitan University for ^129^I and ^137^Cs, respectively. Data were finally compiled at Waseda University. All institutions referred to here are located in Tokyo, Japan.
Data accessibility	Repository name: Mendeley Data Data identification number: 10.17632/fx3j3yph9t. Direct URL to data: https://data.mendeley.com/datasets/fx3j3yph9t/1
Related research article	Mitsuru Ebihara, Naoki Shirai, Yasuji Oura, Haruo Tsuruta, Hiroyuki Matsuzaki, Yuichi Moriguchi, Time-series variations of atmospheric ^129^I concentrations and ^129^I/^137^Cs ratios in suspended particulate matter collected in eastern Japan immediately after the 2011 nuclear accident in Fukushima, Japan, Journal of Environmental Radioactivity 250 (2011) 106,907 (10.1016/j.jenvrad.2022.106907)

## Value of the Data

• These data are useful because they can document the atmospheric concentration of ^131^I [2], which is one of the most concerned radioactive nuclides on the occasion of nuclear accidents and could not be sufficiently quantified due to its short half-life (about 8 days). As ^129^I is an isotope to ^131^I and has a long half-life (1.5 × 10^7^ y), it can be a proxy of ^129^I [2]. These data are also useful for tracing the transfer of radioiodine dispersed into the atmosphere.
• These data benefit atmospheric scientists who manage to model the transportation of radioactive air masses (e.g., [3]). The data also benefit health physicists managing to estimate the radiation exposure due to radionuclides released into the environment (e.g., [4]).
• These data can be used for estimating the degree of radiation exposure against thyroid due to inhalation of aerosol-carrying ^131^I. These data can be also used for chronologically depicting how the nuclear accident was expanded since the nuclear power reactors at FD1NPP were damaged by an earthquake-triggered tsunami in March, 2011.

## 1 Data Description

**Fig. 1:**
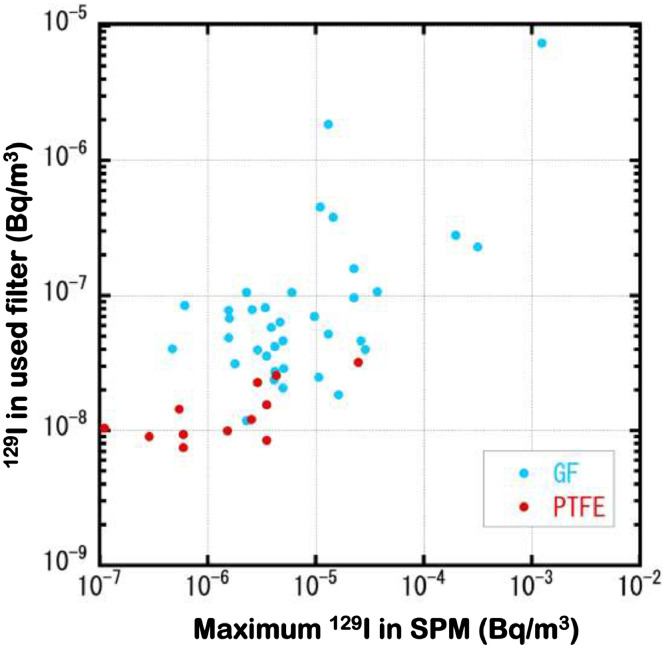
Relationship between ^129^I contents in used-filter blanks (spot blanks) and maximum ^129^I activity concentration (in Ba/m^3^) among SPM samples collected on the same filter roll as sampled for corresponding spot blanks. Spot blank values are shown in Bq/m^3^. These values are derived from the data in Table 4(d) (in Bq) in order to be directly compared to the horizontal axis values (in Bq/m^3^) from Tables 2 and 3. See text for details. Blue and red dot symbols denote activity concentration values derived from SPM samples collected with glass fiber (GF) filter and polytetrafluoroethylene (PTFE) filter, respectively.

This data article presents data of the atmospheric activity concentrations (in Bq/m^3^) of ^129^I dispersed into the environment as aerosol immediately after the nuclear accident at Fukushima Daiichi Nuclear Power Plant on March 11, 2011. Data were obtained by measuring quantities of ^129^I in suspended particulate matter (SPM) collected on filter tapes at 41 SPM monitoring sites in Fukushima and other prefectures in eastern Japan, including the metropolitan area of Tokyo and the surrounding area. In our research project, atmospheric activity concentrations of ^129^I and ^137^Cs and their activity ratios were obtained for 920 SPM samples. Discussion related to some of those samples was made in the research article entitled “Time-series variations of atmospheric ^129^I concentrations and ^129^I/^137^Cs ratios in eastern Japan just after the 2011 nuclear accident in Fukushima, Japan” [1], where 363 sets were presented. The remaining 557 data are presented in this article, so this data article makes up for the original research article [1]. This article contains four tables (Tables 1–4) and one figure (Fig. 1). Table 1 summarizes sampling information for the SPM samples. A similar table appears as Table 1 in the research article [1], but Table 1 of this article provides detailed information. For instance, each location is specified in terms of a set of longitude and latitude. Besides, a flow (suction) late (in L/min) and the number of SPM samples analyzed also are given for each sampling site. A flow rate is from 15 to 18 L/min, with 18 L/min being a standard rate. With one hour suction, a flow rate of 18 L/min corresponds to 1.08 m^3^/h for one SPM sample. Atmospheric SPM samples were collected with two types of filter materials (glass fiber (GF) and polytetrafluoroethylene (PTFE)). Among the 41 sampling sites where the SPM samples were collected, GF and PTFE filters were used at 31 and 10 sites, respectively. Tables 2 and 3 list hourly atmospheric radioactivity concentrations (in Bq/m^3^) of ^129^I and ^137^Cs and their radioactivity ratios (in Bq/Bq) for SPM samples collected with the use of GF filter and PTFE filter, respectively. The activity concentration data of ^137^Cs are from Oura et al. [5] and Tsuruta et al. [6]. Numerical values in Table 2 are given either in bold or in italics. Data in bold were judged to be free from cross contamination of radioactive nuclides and reliable enough for scientific discussion, while those in italics are suspected to be cross-contaminated and should be regarded as inaccurate values. Among the data in Table 2 for 445 SPM samples, 338 and 107 data are in bold and in italics, respectively. In Table 3, where 112 data are presented, besides the above-mentioned two categolized data (accurate and inaccurate data in bold and italics, respectively), the third group of data are indicated in italicized bold for ^129^I activity concentrations and ^129^I/^137^Cs activity ratios of such SPM samples whose ^137^Cs activity data were judged to be accurate (shown in bold), being exempted from cross-contamination. In comparing ^129^I activity concentration values determined from GF filter-collected SPM with those from PTFE-collected samples, it was found that PTFE-collected SPM samples were systematically lower than those from GF-collected SPM samples. Therefore, the third-categolized values in Table 3 were to be regarded as reference values [1]. In Table 4, absolute values of the ^129^I radioactivity (in Bq) for four kinds of blank samples are summarized. These are reagent blanks (a), procedure blanks including reagent blanks (b) and filter blanks. For filter blanks, two kinds of filter blank values were obtained for brand-new filter (c) and used-filter (d). Used-filter samples were taken from open (marginal) space between two sets of consecutive 24 spots (for one day). In Table 4(a) and (b), experimental run numbers (run #), iodine (^127^I) mass used as carriers (in mg) and ^129^I activity per unit mass of iodine (in Bq/mg I) are given. In Table 4(c), in addition to iodine carrier mass (in mg) and ^129^I activity per unit iodine mass (in Bq/mg I), blank values not only for filters used for collecting SPM but also those for collecting APM [2] are given. In Table 4(d), SPM sampling sites, run #, sampling date, carrier mass of iodine (in mg) are given. Values of the ^129^I activity (in Bq) in Table 4(c) and 4(d) include procedural blank values. Fig. 1 shows a relationship between used-filter blanks (spot blanks) of the ^129^I activity (on the vertical axis) and maximum values of the ^129^I activity concentration (in Ba/m^3^; Tables 2 and 3) among SPM samples collected on the same filter roll as sampled for spot blanks (on the horizontal axis). Data are separately plotted for GF filters (in blue) and PTFE filters (in red). Values on the vertical axis are shown in Bq/m^3^, being equivalent to the horizontal axis values. Because a quarter part (∼1 × 1 cm) of each whole SPM sample (∼2 × 2 cm for the filter size) was used for spot blank analysis and each SPM sample contains ^129^I in ∼1 m^3^ air, the vertical axis values are about four times the corresponding values in Table 4(d).

**Table 1:** Sampling sites, collecting conditions and the number of SPM samples analyzed in this study.

Site Code #^a^	Location	Longitude^b^ (East)	Latitude^c^ (North)	Filter material^d^	Flow rate (L/min)	# of samples
Miyagi prefecture						
04-030	Marumori-machi	140.82	37.86	GF	15	26
Fukushima prefecture						
07-002	Fukushima-shi	140.46	37.74	PTFE	18	13
07-003	Fukushima-shi	140.45	37.77	GF	16.7	29
07-004	Fukushima-shi	140.47	37.75	GF	18	32
07-012	Koriyama-shi	140.34	37.39	GF	18	44
07-019	Iwaki-shi	140.90	36.95	GF	18	2
07-020	Iwaki-shi	140.89	36.96	GF	18	7
07-030	Shirakawa-shi	140.22	37.12	GF	16.7	36
07-031	Minamisoma-shi	140.95	37.64	GF	16.7	79
07-032	Sukagawa-shi	140.37	37.29	GF	16.7	38
07-034	Soma-shi	140.92	37.80	PTFE	18	48
07-035	Nihonmatsu-shi	140.46	37.59	GF	18	56
07-036	Minamiaisu-machi	139.78	37.20	GF	15	9
07-037	Yabuki-machi	140.34	37.20	GF	18	44
07-038	Tanakura-machi	140.38	37.02	GF	18	42
07-040	Naraha-machi	140.99	27.27	GF	15	61
07-042	Futaba-machi	141.01	37.44	GF	18	167
07-043	Shinchi-machi	140.91	37.87	GF	18	45
Ibaraki prefecture						
08-009	Tsuchiura-shi	140.19	36.07	GF	18	11
08-010	Tsuchiura-shi	140.17	36.04	GF	18	2
08-011	Koga-shi	139.72	36.20	GF	18	1
08-015	Shimotsuma-shi	139.96	36.18	GF	18	1
08-022	Toride-shi	140.05	35.91	GF	18	7
08-023	Tsukuba-shi	140.02	36.10	GF	18	1
08-032	Kamisu-shi	140.63	35.92	PTFE	16.7	4
08-036	Kamisu-shi	140.70	35.85	PTFE	16.7	4
08-041	Moriya-shi	139.98	35.95	GF	16.7	2
Saitama prefecture						
11-057	Wako-shi	139.62	35.78	GF	18	4
11-064	Misato-shi	139.88	35.84	GF	18	13
Chiba prefecture						
12-004	Chiba-shi	140.14	35.66	GF	18	12
12-027	Choshi-shi	140.82	35.73	PTFE	18	10
12-061	Narita-shi	140.30	35.77	GF	18	9
12-070	Kashiwa-shi	139.96	35.9	GF	18	7
12-084	Ichihara-shi	140.07	35.53	GF	15	4
12-093	Abiko-shi	140.08	35.86	GF	15	3
12-117	Sakae-machi	140.25	35.85	PTFE	18	3
12-118	Narita-shi	140.42	35.85	PTFE	18	10
Tokyo Metropolis						
13-023	Meguro-ku	139.68	35.62	PTFE	18	14
13-025	Meguro-ku	139.68	35.62	GF	15	14
13-069	Machida-shi	139.48	35.59	PTFE	18	2
Kanagawa prefecture						
14-008	Yokohama-shi	139.66	35.41	PTFE	18	4

a Corresponding to the numbers shown in Fig. 1 of Ebihara et al. [1].

b 140.14 stands for 140^0^14′E.

c 35.66 stands for 35^0^66′N.

d GF: Glass fiber filter; PTFE: Polytetrafluoroethylene filter.

**Table 2:** Hourly atmospheric concentrations of ^129^I and ^137^Cs and their activity ratios for SPM samples collected with glass fiber filter tapes^a^.

					^129^I/^137^Cs	

Site code ID #^b^	Sampling date	Sampling time^c^	^129^I (Bq/m^3^)^d^	^137^Cs (Bq/m^3^)^e^	(Bq/Bq)^f^	RE (%)^g^
04-030	March 20	00-01	**9.9E-06 ± 1.7E-07**	**150 ± 3**	**6.6E-08 ± 1.7E-09**	2.5
		13-14	**2.3E-07 ± 7.6E-09**	**1.0 ± 0.1**	**2.3E-07 ± 1.8E-08**	7.9
		14-15	**4.5E-07 ± 1.5E-08**	**4.6 ± 0.1**	**9.8E-08 ± 4.4E-09**	4.5
		15-16	*2.6E-07 ± 8.5E-09*	*1.4 ± 0.1*	*1.9E-07 ± 1.4E-08*	7.5
		16-17	*7.7E-07 ± 2.4E-08*	*5.2 ± 0.2*	*1.5E-07 ± 6.4E-09*	4.3
		17-18	*4.6E-07 ± 1.2E-08*	*2.8 ± 0.1*	*1.6E-07 ± 7.3E-09*	4.5
		18-19	**2.2E-05 ± 3.1E-07**	**188 ± 3**	**1.2E-07 ± 2.7E-09**	2.3
		19-20	**3.7E-05 ± 6.3E-07**	**343 ± 6**	**1.1E-07 ± 2.7E-09**	2.5
		20-21	**2.8E-05 ± 3.9E-07**	**313 ± 6**	**9.0E-08 ± 2.0E-09**	2.2
		21-22	**3.1E-05 ± 5.3E-07**	**263 ± 5**	**1.2E-07 ± 2.9E-09**	2.5
		22-23	**2.2E-05 ± 3.1E-07**	**192 ± 4**	**1.1E-07 ± 2.6E-09**	2.3
		23-24	**1.1E-05 ± 1.9E-07**	**145 ± 3**	**7.6E-08 ± 1.9E-09**	2.5
	March 21	00-01	**5.6E-06 ± 1.0E-07**	**80.7 ± 1.5**	**6.9E-08 ± 1.8E-09**	2.6
		01-02	**3.8E-06 ± 6.6E-08**	**65.8 ± 1.2**	**5.8E-08 ± 1.5E-09**	2.5
		02-03	**2.0E-06 ± 4.4E-08**	**36.5 ± 0.8**	**5.4E-08 ± 1.6E-09**	3.0
		03-04	**1.6E-06 ± 3.0E-08**	**24.6 ± 0.6**	**6.4E-08 ± 1.9E-09**	3.0
		04-05	**7.6E-07 ± 2.2E-08**	**11.5 ± 0.3**	**6.6E-08 ± 2.5E-09**	3.7
		05-06	**1.0E-06 ± 2.7E-08**	**11.0 ± 0.3**	**9.4E-08 ± 3.4E-09**	3.6
		06-07	**8.7E-07 ± 2.1E-08**	**10.6 ± 0.3**	**8.1E-08 ± 2.7E-09**	3.4
		07-08	*1.4E-05 ± 2.3E-07*	*13.2 ± 0.4*	*1.1E-06 ± 3.3E-08*	3.2
		08-09	*1.9E-05 ± 3.6E-07*	*11.3 ± 0.3*	*1.7E-06 ± 5.7E-08*	3.4
		09-10	*1.6E-05 ± 2.6E-07*	*14.7 ± 0.3*	*1.1E-06 ± 3.0E-08*	2.8
		10-11	*1.1E-05 ± 2.2E-07*	*10.5 ± 0.3*	*1.1E-06 ± 3.6E-08*	3.4
		11-12	*7.1E-06 ± 1.2E-07*	*6.0 ± 0.2*	*1.2E-06 ± 4.7E-08*	4.0
		12-13	*5.0E-06 ± 1.0E-07*	*4.3 ± 0.2*	*1.2E-06 ± 4.8E-08*	4.1
		13-14	*4.6E-06 ± 8.4E-08*	*4.1 ± 0.1*	*1.1E-06 ± 4.4E-08*	3.9
						
07-003	March 15	17-18	**9.4E-07 ± 2.3E-08**	**7.0 ± 0.3**	**1.4E-07 ± 6.1E-09**	4.5
		18-19	**1.6E-06 ± 3.0E-08**	**6.3 ± 0.3**	**2.5E-07 ± 1.1E-08**	4.5
		19-20	**1.5E-06 ± 2.8E-08**	**8.8 ± 0.3**	**1.7E-07 ± 7.4E-09**	4.3
		20-21	**2.1E-06 ± 3.9E-08**	**11.8 ± 0.4**	**1.7E-07 ± 7.4E-09**	4.2
		21-22	**2.7E-06 ± 4.8E-08**	**27.3 ± 1.0**	**9.9E-08 ± 3.9E-09**	4.0
		22-23	**5.1E-07 ± 1.1E-08**	**4.1 ± 0.6**	**1.2E-07 ± 1.9E-08**	15
		23-24	*1.4E-07 ± 4.5E-09*	*0.28 ± 0.04*	*5.1E-07 ± 8.4E-08*	16
	March 16	00-01	**1.7E-06 ± 3.1E-08**	**10.3 ± 0.4**	**1.6E-07 ± 6.9E-09**	4.2
		01-02	**1.8E-06 ± 4.0E-08**	**11.4 ± 0.4**	**1.6E-07 ± 6.2E-09**	4.0
		02-03	**8.7E-07 ± 2.1E-08**	**4.3 ± 0.2**	**2.0E-07 ± 9.7E-09**	4.9
	March 20	13-14	**3.3E-07 ± 1.2E-08**	**1.5 ± 0.1**	**2.3E-07 ± 2.3E-08**	10
		14-15	**2.5E-06 ± 5.9E-08**	**41.1 ± 1.3**	**6.2E-08 ± 2.4E-09**	3.9
		15-16	**2.5E-06 ± 5.5E-08**	**37.2 ± 1.3**	**6.7E-08 ± 2.8E-09**	4.2
		16-17	**1.5E-06 ± 4.0E-08**	**20.8 ± 0.7**	**7.4E-08 ± 3.1E-09**	4.2
		17-18	**1.4E-06 ± 3.0E-08**	**19.9 ± 0.7**	**7.0E-08 ± 2.8E-09**	4.0
		18-19	**1.6E-06 ± 4.8E-08**	**20.6 ± 0.8**	**7.8E-08 ± 3.7E-09**	4.8
		19-20	**1.4E-06 ± 3.3E-08**	**16.3 ± 0.6**	**8.7E-08 ± 4.0E-09**	4.6
		20-21	**1.3E-06 ± 3.1E-08**	**15.1 ± 0.6**	**9.0E-08 ± 3.9E-09**	4.3
		21-22	**1.4E-06 ± 3.1E-08**	**15.3 ± 0.6**	**9.1E-08 ± 3.9E-09**	4.3
		22-23	**2.2E-06 ± 4.8E-08**	**24.1 ± 0.8**	**9.1E-08 ± 3.6E-09**	3.9
		23-24	**2.5E-06 ± 5.5E-08**	**27.8 ± 0.9**	**9.0E-08 ± 3.6E-09**	4.0
	March 21	00-01	**2.3E-06 ± 5.1E-08**	**30.8 ± 1.0**	**7.4E-08 ± 2.9E-09**	3.9
		01-02	**2.6E-06 ± 9.6E-08**	**33.1 ± 1.1**	**7.9E-08 ± 3.9E-09**	5.0
		02-03	**2.6E-06 ± 5.8E-08**	**37.2 ± 1.3**	**7.1E-08 ± 2.9E-09**	4.1
		03-04	**2.7E-06 ± 5.9E-08**	**33.5 ± 1.2**	**8.0E-08 ± 3.3E-09**	4.1
		04-05	**5.0E-06 ± 1.1E-07**	**31.2 ± 1.0**	**1.6E-07 ± 6.2E-09**	3.9
		05-06	**1.9E-06 ± 4.1E-08**	**26.6 ± 0.9**	**7.0E-08 ± 2.9E-09**	4.2
		06-07	*4.2E-07 ± 9.0E-09*	*0.49 ± 0.04*	*8.6E-07 ± 7.2E-08*	8.4
		07-08	*1.5E-07 ± 4.4E-09*	*0.26 ± 0.02*	*5.7E-07 ± 5.4E-08*	9.5
07-004	March 15	16-17	**2.0E-07 ± 4.7E-09**	**0.58 ± 0.13**	**3.3E-07 ± 7.3E-08**	22
		17-18	**9.3E-07 ± 1.5E-08**	**3.4 ± 0.3**	**2.7E-07 ± 2.2E-08**	7.9
		18-19	**1.5E-06 ± 3.3E-08**	**7.0 ± 0.4**	**2.2E-07 ± 1.3E-08**	5.9
		19-20	**1.9E-06 ± 3.9E-08**	**8.8 ± 0.4**	**2.1E-07 ± 1.2E-08**	5.5
		20-21	**2.8E-06 ± 5.8E-08**	**20.2 ± 0.8**	**1.4E-07 ± 6.0E-09**	4.3
		21-22	**3.6E-06 ± 6.9E-08**	**23.0 ± 0.8**	**1.6E-07 ± 6.5E-09**	4.1
		22-23	**8.2E-07 ± 2.2E-07**	**6.0 ± 0.4**	**1.4E-07 ± 3.8E-08**	28
		23-24	**3.0E-07 ± 5.9E-09**	**1.1 ± 0.2**	**2.7E-07 ± 3.8E-08**	14
	March 16	00-01	*3.0E-07 ± 5.9E-09*	*1.3 ± 0.2*	*2.3E-07 ± 3.0E-08*	13
		01-02	**9.1E-07 ± 2.3E-08**	**4.6 ± 0.3**	**2.0E-07 ± 1.4E-08**	7.2
		02-03	**5.6E-07 ± 1.0E-08**	**3.1 ± 0.2**	**1.8E-07 ± 1.5E-08**	8.2
	March 20	13-14	**2.6E-07 ± 6.7E-09**	**2.4 ± 0.2**	**1.1E-07 ± 1.1E-08**	9.7
		14-15	**9.7E-06 ± 2.1E-07**	**53.2 ± 1.6**	**1.8E-07 ± 6.7E-09**	3.7
		15-16	**8.7E-06 ± 2.7E-07**	**39.5 ± 1.3**	**2.2E-07 ± 9.9E-09**	4.5
		16-17	**1.4E-06 ± 2.9E-08**	**15.1 ± 0.6**	**9.0E-08 ± 4.2E-09**	4.7
		17-18	**1.3E-06 ± 2.6E-08**	**11.8 ± 0.5**	**1.1E-07 ± 5.4E-09**	5.0
		18-19	**2.8E-06 ± 5.7E-08**	**19.7 ± 0.8**	**1.4E-07 ± 6.2E-09**	4.3
		19-20	**2.0E-06 ± 4.0E-08**	**23.2 ± 0.8**	**8.7E-08 ± 3.6E-09**	4.2
		20-21	**1.5E-06 ± 2.9E-08**	**10.6 ± 0.5**	**1.4E-07 ± 7.0E-09**	5.1
		21-22	**8.4E-07 ± 1.9E-08**	**10.6 ± 0.5**	**7.9E-08 ± 4.1E-09**	5.2
		22-23	**9.5E-07 ± 2.0E-08**	**11.5 ± 0.5**	**8.3E-08 ± 4.2E-09**	5.0
	March 21	23-24	**1.2E-06 ± 2.5E-08**	**18.7 ± 0.7**	**6.4E-08 ± 2.8E-09**	4.4
		00-01	**1.5E-06 ± 2.9E-08**	**26.1 ± 0.9**	**5.8E-08 ± 2.3E-09**	4.0
		01-02	**1.8E-06 ± 3.5E-08**	**34.2 ± 1.1**	**5.3E-08 ± 2.0E-09**	3.8
		02-03	**2.1E-06 ± 4.4E-08**	**35.8 ± 1.2**	**6.0E-08 ± 2.3E-09**	3.9
		03-04	**2.1E-06 ± 4.1E-08**	**33.7 ± 1.1**	**6.2E-08 ± 2.4E-09**	3.8
		04-05	**2.0E-06 ± 4.0E-08**	**33.5 ± 1.1**	**6.1E-08 ± 2.3E-09**	3.8
		05-06	**1.8E-06 ± 3.7E-08**	**31.0 ± 1.0**	**5.8E-08 ± 2.3E-09**	3.9
		06-07	**4.9E-07 ± 1.1E-08**	**5.4 ± 0.3**	**9.1E-08 ± 6.0E-09**	6.6
		07-08	*1.0E-07 ± 4.2E-09*	*0.39 ± 0.10*	*2.6E-07 ± 6.7E-08*	26
		08-09	*3.5E-07 ± 6.3E-09*	*0.41 ± 0.10*	*8.6E-07 ± 2.1E-07*	25
		09-10	*1.2E-06 ± 2.0E-08*	*0.47 ± 0.11*	*2.5E-06 ± 5.9E-07*	23
						
07-012	March 15	13-14	**5.3E-07 ± 1.1E-08**	**8.6 ± 0.5**	**6.1E-08 ± 3.6E-09**	5.9
		14-15	**1.9E-06 ± 3.9E-08**	**34.3 ± 1.2**	**5.7E-08 ± 2.2E-09**	3.9
		15-16	**4.0E-07 ± 9.0E-09**	**3.5 ± 0.3**	**1.2E-07 ± 1.1E-08**	9.3
		16-17	**3.8E-07 ± 8.6E-09**	**8.21 ± 0.47**	**4.6E-08 ± 2.8E-09**	6.1
		17-18	**1.4E-07 ± 4.0E-09**	**3.4 ± 0.3**	**4.2E-08 ± 4.0E-09**	9.4
		18-19	**1.3E-07 ± 3.3E-09**	**2.3 ± 0.3**	**5.4E-08 ± 6.3E-09**	12
		19-20	**1.3E-07 ± 3.6E-09**	**2.7 ± 0.3**	**5.0E-08 ± 5.3E-09**	11
		20-21	**1.3E-07 ± 3.6E-09**	**3.1 ± 0.3**	**4.3E-08 ± 4.2E-09**	9.9
		21-22	**1.2E-07 ± 4.4E-09**	**2.1 ± 0.3**	**5.8E-08 ± 7.5E-09**	13
		22-23	**9.6E-08 ± 3.4E-09**	**2.3 ± 0.3**	**4.2E-08 ± 5.0E-09**	12
		23-24	**1.0E-07 ± 3.5E-09**	**1.5 ± 0.2**	**6.9E-08 ± 1.1E-08**	15
	March 16	00-01	**4.2E-07 ± 8.4E-09**	**1.8 ± 0.2**	**2.4E-07 ± 3.2E-08**	14
		01-02	**5.0E-07 ± 8.9E-09**	**2.1 ± 0.3**	**2.4E-07 ± 3.1E-08**	13
		02-03	**6.3E-07 ± 1.2E-08**	**1.2 ± 0.2**	**5.3E-07 ± 9.5E-08**	18
		06-07	*3.4E-07 ± 2.2E-08*	*0.65 ± 0.19*	*5.2E-07 ± 1.6E-07*	30
		07-08	*7.3E-08 ± 5.6E-09*	*0.49 ± 0.18*	*1.5E-07 ± 5.6E-08*	37
	March 20	14-15	**8.8E-08 ± 3.3E-09**	**3.1 ± 0.3**	**2.9E-08 ± 3.0E-09**	10
		15-16	**8.0E-08 ± 2.8E-09**	**1.7 ± 0.2**	**4.8E-08 ± 7.0E-09**	15
		16-17	**1.3E-07 ± 3.6E-09**	**3.2 ± 0.3**	**4.0E-08 ± 3.9E-09**	9.7
		17-18	**2.0E-07 ± 4.7E-09**	**4.1 ± 0.3**	**4.9E-08 ± 4.1E-09**	8.4
		18-19	**1.6E-07 ± 3.8E-09**	**2.8 ± 0.3**	**5.8E-08 ± 6.0E-09**	10
		19-20	**1.4E-07 ± 3.7E-09**	**3.3 ± 0.3**	**4.3E-08 ± 4.1E-09**	9.6
		20-21	**2.2E-06 ± 4.4E-08**	**33.7 ± 1.1**	**6.5E-08 ± 2.5E-09**	3.9
		21-22	**2.9E-06 ± 5.7E-08**	**54.7 ± 1.6**	**5.3E-08 ± 1.9E-09**	3.6
		22-23	**3.8E-06 ± 7.4E-08**	**84.9 ± 2.4**	**4.5E-08 ± 1.5E-09**	3.4
		23-24	**2.8E-06 ± 5.6E-08**	**63.2 ± 1.8**	**4.5E-08 ± 1.6E-09**	3.5
	March 21	00-01	**2.3E-06 ± 4.8E-08**	**52.6 ± 1.6**	**4.5E-08 ± 1.6E-09**	3.7
		01-02	**2.5E-06 ± 4.9E-08**	**59.1 ± 1.8**	**4.2E-08 ± 1.5E-09**	3.6
		02-03	**2.4E-06 ± 6.4E-08**	**73.1 ± 2.1**	**3.3E-08 ± 1.3E-09**	3.9
		03-04	**2.7E-06 ± 5.4E-08**	**45.3 ± 1.4**	**6.0E-08 ± 2.2E-09**	3.7
		04-05	**1.1E-07 ± 3.4E-09**	**1.6 ± 0.2**	**7.1E-08 ± 1.1E-08**	15
		05-06	**8.1E-08 ± 2.9E-09**	**1.3 ± 0.2**	**6.2E-08 ± 1.1E-08**	17
		06-07	*1.5E-08 ± 4.0E-09*	*0.30 ± 0.17*	*5.2E-08 ± 3.2E-08*	62
		07-08	**3.7E-08 ± 2.2E-09**	**0.80 ± 0.20**	**4.6E-08 ± 1.2E-08**	26
		08-09	**5.9E-08 ± 2.2E-09**	**0.81 ± 0.20**	**7.2E-08 ± 1.8E-08**	25
		12-13	*2.2E-08 ± 5.9E-09*	*0.46 ± 0.17*	*4.7E-08 ± 2.2E-08*	46
		13-14	*1.4E-07 ± 9.8E-09*	*0.71 ± 0.19*	*1.9E-07 ± 5.3E-08*	28
		14-15	*3.6E-07 ± 2.8E-08*	*0.76 ± 0.20*	*4.8E-07 ± 1.3E-07*	27
		15-16	*2.7E-07 ± 5.7E-09*	*1.0 ± 0.2*	*2.7E-07 ± 5.4E-08*	20
		16-17	*3.2E-07 ± 2.0E-08*	*0.73 ± 0.19*	*4.4E-07 ± 1.2E-07*	27
		18-19	*1.1E-07 ± 3.0E-09*	*0.64 ± 0.19*	*1.7E-07 ± 4.9E-08*	29
		19-20	*2.5E-07 ± 5.1E-09*	*0.83 ± 0.19*	*3.0E-07 ± 7.1E-08*	23
		20-21	*2.8E-07 ± 6.8E-09*	*1.3 ± 0.2*	*2.1E-07 ± 3.6E-08*	17
		21-22	*2.7E-07 ± 5.5E-09*	*1.0 ± 0.2*	*2.6E-07 ± 5.2E-08*	20
						
07-019	March 22	12-13	**5.3E-07 ± 1.5E-08**	**1.3 ± 0.2**	**4.2E-07 ± 6.7E-08**	16
		16-17	**3.4E-06 ± 7.4E-08**	**3.7 ± 0.3**	**9.2E-07 ± 7.9E-08**	8.6
						
07-020	March 22	11-12	*1.2E-06 ± 3.0E-08*	*0.57 ± 0.17*	*2.2E-06 ± 6.4E-07*	29
		12-13	*4.2E-06 ± 9.2E-08*	*1.7 ± 0.2*	*2.4E-06 ± 3.2E-07*	13
		15-16	*3.8E-07 ± 1.1E-08*	*1.2 ± 0.2*	*3.3E-07 ± 5.6E-08*	17
		16-17	*1.1E-05 ± 2.3E-07*	*5.9 ± 0.4*	*1.9E-06 ± 1.3E-07*	6.9
		17-18	*3.9E-07 ± 1.2E-08*	*0.32 ± 0.15*	*1.2E-06 ± 5.8E-07*	47
		21-22	*1.6E-07 ± 7.0E-09*	*1.1 ± 0.2*	*1.4E-07 ± 2.7E-08*	19
		22-23	*3.5E-07 ± 1.1E-08*	*0.52 ± 0.16*	*6.7E-07 ± 2.1E-07*	31
						
07-030	March 15	12-13	**3.2E-06 ± 7.2E-08**	**32.7 ± 1.1**	**9.9E-08 ± 4.1E-09**	4.1
		13-14	**1.1E-05 ± 2.5E-07**	**113 ± 3**	**9.4E-08 ± 3.4E-09**	3.6
		14-15	**5.3E-06 ± 1.2E-07**	**60.8 ± 1.8**	**8.7E-08 ± 3.2E-09**	3.7
		15-16	**2.3E-06 ± 6.1E-08**	**13.3 ± 0.6**	**1.7E-07 ± 9.3E-09**	5.4
		16-17	**7.2E-07 ± 2.2E-08**	**7.2 ± 0.5**	**1.0E-07 ± 7.0E-09**	7.0
		17-18	**7.3E-07 ± 4.3E-07**	**6.7 ± 0.4**	**1.1E-07 ± 6.4E-08**	59
		18-19	**7.2E-07 ± 2.4E-08**	**9.2 ± 0.5**	**7.8E-08 ± 5.1E-09**	6.5
		19-20	**8.6E-07 ± 2.4E-08**	**9.8 ± 0.5**	**8.8E-08 ± 5.3E-09**	6.1
		20-21	**8.8E-07 ± 2.4E-08**	**9.4 ± 0.5**	**9.4E-08 ± 5.7E-09**	6.1
		21-22	**8.6E-07 ± 2.8E-08**	**12.4 ± 0.6**	**7.0E-08 ± 4.1E-09**	5.9
		22-23	**4.5E-07 ± 1.4E-08**	**3.6 ± 0.3**	**1.2E-07 ± 1.2E-08**	9.4
		23-24	*3.2E-07 ± 1.1E-08*	*2.5 ±* 0.3	*1.3E-07 ± 1.5E-08*	12
	March 16	00-01	*3.1E-06 ± 7.4E-08*	*2.6 ± 0.3*	*1.2E-06 ± 1.3E-07*	11
		01-02	*1.1E-06 ± 2.8E-08*	*0.75 ± 0.19*	*1.4E-06 ± 3.7E-07*	26
		02-03	*3.8E-07 ± 1.3E-08*	*0.36 ± 0.17*	*1.1E-06 ± 4.9E-07*	46
		03-04	*5.8E-07 ± 1.6E-08*	*0.61 ± 0.19*	*9.6E-07 ± 3.0E-07*	31
		04-05	*3.0E-07 ± 1.0E-08*	*< 0.3*		
		05-06	*1.2E-07 ± 7.1E-09*	*< 0.3*		
	March 20	14-15	**3.2E-07 ± 1.2E-08**	**1.5 ± 0.2**	**2.2E-07 ± 3.6E-08**	16
		15-16	**2.4E-07 ± 8.6E-09**	**2.6 ± 0.3**	**9.3E-08 ± 1.1E-08**	11
		16-17	**2.6E-07 ± 9.8E-09**	**2.3 ± 0.3**	**1.1E-07 ± 1.4E-08**	12
		17-18	**4.0E-07 ± 1.4E-08**	**5.0 ± 0.4**	**8.1E-08 ± 6.6E-09**	8.2
		18-19	**4.8E-07 ± 1.4E-08**	**5.2 ± 0.4**	**9.2E-08 ± 7.3E-09**	7.9
		19-20	**5.8E-07 ± 1.7E-08**	**5.9 ± 0.4**	**9.9E-08 ± 7.4E-09**	7.4
		20-21	**5.4E-07 ± 3.6E-08**	**5.5 ± 0.4**	**9.9E-08 ± 9.6E-09**	9.7
		21-22	**5.7E-07 ± 1.7E-08**	**6.4 ± 0.4**	**9.0E-08 ± 6.5E-09**	7.2
		22-23	**5.0E-07 ± 1.5E-08**	**5.7 ± 0.4**	**8.8E-08 ± 6.7E-09**	7.6
		23-24	**4.5E-07 ± 1.4E-08**	**6.2 ± 0.4**	**7.2E-08 ± 5.3E-09**	7.3
	March 21	00-01	**1.5E-06 ± 3.7E-08**	**15.3 ± 0.7**	**9.6E-08 ± 4.9E-09**	5.1
		01-02	**2.4E-06 ± 5.9E-08**	**25.8 ± 1.0**	**9.5E-08 ± 4.2E-09**	4.4
		02-03	**3.3E-06 ± 7.7E-08**	**31.4 ± 1.1**	**1.1E-07 ± 4.5E-09**	4.2
		03-04	**2.9E-06 ± 6.8E-08**	**25.2 ± 0.9**	**1.1E-07 ± 5.0E-09**	4.4
		04-05	**2.4E-06 ± 5.6E-08**	**25.1 ± 0.9**	**9.6E-08 ± 4.2E-09**	4.4
		05-06	**2.5E-06 ± 5.9E-08**	**28.9 ± 1.0**	**8.7E-08 ± 3.7E-09**	4.3
		06-07	**1.5E-06 ± 3.7E-08**	**15.4 ± 0.7**	**9.8E-08 ± 5.0E-09**	5.1
		07-08	**4.9E-07 ± 1.8E-08**	**3.8 ± 0.3**	**1.3E-07 ± 1.2E-08**	9.4
						
07-032	March 15	12-13	**3.8E-06 ± 1.8E-07**	**23.8 ± 0.9**	**1.6E-07 ± 9.8E-09**	6.1
		13-14	**2.9E-05 ± 6.1E-07**	**271 ± 7**	**1.1E-07 ± 3.5E-09**	3.3
		14-15	**4.3E-06 ± 9.3E-08**	**42.7 ± 1.4**	**1.0E-07 ± 3.9E-09**	3.9
		15-16	**1.4E-06 ± 3.5E-08**	**9.5 ± 0.5**	**1.4E-07 ± 8.8E-09**	6.1
		16-17	**5.5E-07 ± 1.6E-08**	**6.6 ± 0.4**	**8.3E-08 ± 5.8E-09**	7.1
		17-18	**3.2E-07 ± 1.2E-08**	**2.5 ± 0.3**	**1.3E-07 ± 1.5E-08**	12
		20-21	*1.0E-06 ± 6.1E-08*	*1.1 ± 0.2*	*8.9E-07 ± 1.8E-07*	20
		21-22	*1.2E-05 ± 5.9E-07*	*2.9 ± 0.3*	*4.0E-06 ± 4.5E-07*	11
		22-23	*1.4E-06 ± 7.7E-08*	*0.67 ± 0.19*	*2.1E-06 ± 6.1E-07*	29
		23-24	*3.2E-07 ± 2.0E-08*	*0.53 ± 0.19*	*6.1E-07 ± 2.2E-07*	36
	March 16	00-01	*2.6E-07 ± 1.6E-08*	*0.41 ± 0.17*	*6.4E-07 ± 2.7E-07*	42
		01-02	*1.4E-06 ± 7.6E-08*	*1.9 ± 0.2*	*7.5E-07 ± 1.1E-07*	14
		02-03	*1.2E-06 ± 6.8E-08*	*0.88 ± 0.21*	*1.4E-06 ± 3.4E-07*	25
	March 20	13-14	**2.3E-07 ± 7.8E-09**	**0.71 ± 0.18**	**3.2E-07 ± 8.5E-08**	26
		14-15	**1.9E-07 ± 8.2E-09**	**1.3 ± 0.2**	**1.4E-07 ± 2.5E-08**	17
		15-16	**2.5E-07 ± 8.1E-09**	**1.4 ± 0.2**	**1.7E-07 ± 2.8E-08**	16
		16-17	**3.5E-07 ± 1.0E-08**	**3.4 ± 0.3**	**1.0E-07 ± 9.6E-09**	9.5
		17-18	**3.3E-07 ± 9.5E-09**	**2.9 ± 0.3**	**1.1E-07 ± 1.2E-08**	10
		18-19	**3.3E-07 ± 9.6E-09**	**3.1 ± 0.3**	**1.0E-07 ± 1.1E-08**	10
		19-20	**3.9E-07 ± 1.2E-08**	**3.6 ± 0.3**	**1.1E-07 ± 1.0E-08**	9.4
		20-21	**3.4E-07 ± 1.1E-08**	**3.0 ± 0.3**	**1.1E-07 ± 1.2E-08**	10
		21-22	**1.0E-06 ± 2.4E-08**	**10.3 ± 0.5**	**9.8E-08 ± 5.7E-09**	5.8
		22-23	**4.1E-06 ± 8.9E-08**	**40.4 ± 1.3**	**1.0E-07 ± 4.0E-09**	3.9
		23-24	**4.8E-06 ± 1.0E-07**	**53.8 ± 1.6**	**9.0E-08 ± 3.3E-09**	3.7
	March 21	00-01	**4.7E-06 ± 9.5E-08**	**53.1 ± 1.6**	**8.9E-08 ± 3.3E-09**	3.7
		01-02	**3.9E-06 ± 7.9E-08**	**46.9 ± 1.5**	**8.3E-08 ± 3.1E-09**	3.8
		02-03	**3.9E-06 ± 8.0E-08**	**42.9 ± 1.4**	**9.1E-08 ± 3.5E-09**	3.8
		03-04	**4.0E-06 ± 8.2E-08**	**41.2 ± 1.3**	**9.6E-08 ± 3.7E-09**	3.9
		04-05	**2.2E-06 ± 5.0E-08**	**20.7 ± 0.8**	**1.1E-07 ± 4.9E-09**	4.6
		05-06	**1.2E-06 ± 3.5E-08**	**12.3 ± 0.6**	**9.5E-08 ± 5.4E-09**	5.7
		06-07	**4.4E-07 ± 1.5E-08**	**2.4 ± 0.3**	**1.8E-07 ± 2.3E-08**	12
		07-08	**5.4E-07 ± 1.5E-08**	**4.3 ± 0.4**	**1.2E-07 ± 1.1E-08**	8.6
		08-09	**2.6E-07 ± 9.7E-09**	**2.2 ± 0.3**	**1.2E-07 ± 1.5E-08**	13
		09-10	**4.6E-07 ± 1.2E-08**	**0.74 ± 0.19**	**6.3E-07 ± 1.7E-07**	26
		10-11	*4.7E-07 ± 1.1E-08*	*< 0.3*		
		11-12	*1.6E-06 ± 2.9E-08*	*< 0.3*		
		12-13	*2.4E-08 ± 6.4E-09*	*< 0.3*		
		13-14	*3.2E-07 ± 8.3E-09*	*< 0.3*		
						
07-036	March 15	15-16	**1.7E-07 ± 6.4E-09**	**1.4 ± 0.2**	**1.2E-07 ± 1.6E-08**	14
		16-17	**8.3E-07 ± 1.4E-08**	**5.1 ± 0.4**	**1.6E-07 ± 1.2E-08**	7.1
		17-18	**1.8E-06 ± 2.9E-08**	**12.5 ± 0.6**	**1.5E-07 ± 7.4E-09**	5.0
		18-19	**2.7E-06 ± 4.7E-08**	**9.6 ± 0.5**	**2.8E-07 ± 1.6E-08**	5.5
		19-20	**1.4E-06 ± 2.4E-08**	**4.9 ± 0.3**	**2.9E-07 ± 2.0E-08**	7.2
		20-21	**5.8E-07 ± 1.1E-08**	**3.2 ± 0.3**	**1.9E-07 ± 1.7E-08**	9.0
		21-22	**3.2E-07 ± 8.4E-09**	**1.4 ± 0.2**	**2.2E-07 ± 2.9E-08**	13
		22-23	**1.8E-07 ± 6.9E-09**	**1.4 ± 0.2**	**1.3E-07 ± 1.7E-08**	13
		23-24	**1.9E-07 ± 6.4E-09**	**1.7 ± 0.2**	**1.1E-07 ± 1.3E-08**	12
						
07-037	March 15	11-12	**2.5E-07 ± 8.7E-09**	**1.3 ± 0.2**	**2.0E-07 ± 2.6E-08**	13
		12-13	**1.6E-05 ± 3.6E-07**	**330 ± 8**	**4.9E-08 ± 1.6E-09**	3.4
		13-14	**3.8E-06 ± 1.1E-07**	**92.8 ± 2.6**	**4.1E-08 ± 1.6E-09**	4.0
		14-15	**1.2E-06 ± 3.1E-08**	**22.0 ± 0.8**	**5.5E-08 ± 2.5E-09**	4.5
		15-16	**2.5E-07 ± 9.1E-09**	**4.9 ± 0.3**	**5.0E-08 ± 3.7E-09**	7.4
		16-17	**3.2E-07 ± 1.1E-08**	**6.0 ± 0.4**	**5.3E-08 ± 3.6E-09**	6.8
		17-18	**3.0E-07 ± 1.1E-08**	**5.8 ± 0.3**	**5.1E-08 ± 3.6E-09**	7.1
		18-19	**1.6E-07 ± 7.1E-09**	**1.8 ± 0.2**	**9.0E-08 ± 1.0E-08**	12
		19-20	**1.4E-07 ± 5.8E-09**	**1.6 ± 0.2**	**9.3E-08 ± 1.1E-08**	12
		20-21	**6.3E-08 ± 3.2E-09**	**1.0 ± 0.1**	**6.2E-08 ± 9.6E-09**	16
		21-22	**4.3E-08 ± 2.6E-09**	**0.53 ± 0.11**	**8.1E-08 ± 1.7E-08**	21
		22-23	*2.9E-08 ± 6.0E-09*	*0.22 ± 0.09*	*1.3E-07 ± 5.6E-08*	44
		23-24	*1.7E-08 ± 4.4E-09*	*0.21 ± 0.08*	*8.1E-08 ± 3.7E-08*	46
	March 16	00-01	*1.4E-08 ± 4.4E-09*	*0.19 ± 0.08*	*7.5E-08 ± 3.9E-08*	51
		01-02	*1.0E-07 ± 3.8E-09*	*0.61 ± 0.12*	*1.7E-07 ± 3.2E-08*	19
		02-03	**4.6E-07 ± 9.2E-09**	**1.6 ± 0.2**	**2.9E-07 ± 3.4E-08**	12
		03-04	*1.3E-07 ± 4.1E-09*	*0.26 ± 0.09*	*5.0E-07 ± 1.8E-07*	37
		04-05	*3.4E-08 ± 5.2E-09*	*0.19 ± 0.07*	*1.8E-07 ± 7.4E-08*	41
		05-06	*3.7E-08 ± 5.2E-09*	*0.14 ± 0.07*	*2.6E-07 ± 1.3E-07*	49
		09-10	*8.3E-08 ± 5.2E-09*	*3.6 ± 0.3*	*2.3E-08 ± 2.3E-09*	9.8
		10-11	*9.9E-07 ± 1.8E-08*	*8.0 ± 0.4*	*1.2E-07 ± 6.8E-09*	5.5
		11-12	*2.0E-06 ± 3.2E-08*	*4.0 ± 0.3*	*5.0E-07 ± 3.7E-08*	7.3
		12-13	*5.2E-07 ± 9.7E-09*	*1.4 ± 0.2*	*3.6E-07 ± 4.5E-08*	12
	March 20	13-14	*4.9E-08 ± 4.6E-09*	*0.31 ± 0.11*	*1.6E-07 ± 5.9E-08*	37
		14-15	**1.0E-07 ± 6.1E-09**	**2.6 ± 0.2**	**4.0E-08 ± 4.1E-09**	10
		15-16	**1.0E-07 ± 5.3E-09**	**1.8 ± 0.2**	**5.8E-08 ± 6.9E-09**	12
		16-17	**1.6E-07 ± 4.5E-09**	**3.1 ± 0.3**	**5.0E-08 ± 4.3E-09**	8.6
		17-18	**1.4E-07 ± 4.6E-09**	**2.4 ± 0.2**	**5.6E-08 ± 5.4E-09**	9.6
		18-19	**1.3E-07 ± 4.5E-09**	**2.2 ± 0.2**	**5.9E-08 ± 5.9E-09**	10
		19-20	**1.2E-07 ± 4.2E-09**	**2.2 ± 0.2**	**5.6E-08 ± 5.7E-09**	10
		20-21	**1.2E-07 ± 3.6E-09**	**1.8 ± 0.2**	**6.7E-08 ± 7.3E-09**	11
		21-22	**1.4E-07 ± 5.0E-09**	**1.8 ± 0.2**	**7.9E-08 ± 8.8E-09**	11
		22-23	**2.9E-07 ± 7.3E-09**	**4.5 ± 0.3**	**6.5E-08 ± 4.7E-09**	7.2
		23-24	**1.7E-06 ± 4.6E-08**	**24.6 ± 0.9**	**7.0E-08 ± 3.1E-09**	4.5
	March 21	00-01	**2.1E-06 ± 5.2E-08**	**36.3 ± 1.2**	**5.8E-08 ± 2.4E-09**	4.1
		01-02	**1.6E-06 ± 3.1E-08**	**21.0 ± 0.8**	**7.7E-08 ± 3.2E-09**	4.2
		02-03	**9.8E-07 ± 1.9E-08**	**17.2 ± 0.7**	**5.7E-08 ± 2.5E-09**	4.4
		03-04	**1.1E-06 ± 2.2E-08**	**18.4 ± 0.7**	**5.8E-08 ± 2.6E-09**	4.4
		04-05	**7.3E-07 ± 1.6E-08**	**11.7 ± 0.5**	**6.2E-08 ± 3.2E-09**	5.0
		05-06	**6.7E-07 ± 1.4E-08**	**10.3 ± 0.5**	**6.5E-08 ± 3.4E-09**	5.2
		06-07	**3.1E-07 ± 7.0E-09**	**3.5 ± 0.3**	**8.9E-08 ± 6.9E-09**	7.8
		07-08	**2.1E-07 ± 4.4E-09**	**2.8 ± 0.2**	**7.6E-08 ± 6.5E-09**	8.5
		08-09	**6.9E-08 ± 3.2E-09**	**0.91 ± 0.14**	**7.5E-08 ± 1.2E-08**	16
		09-10	**5.5E-08 ± 3.2E-09**	**1.9 ± 0.2**	**2.9E-08 ± 3.5E-09**	12
07-038	March 15	08-09	**4.3E-07 ± 1.2E-08**	**10.2 ± 0.5**	**4.2E-08 ± 2.3E-09**	5.5
		09-10	**8.3E-07 ± 2.3E-08**	**25.8 ± 0.9**	**3.2E-08 ± 1.4E-09**	4.5
		10-11	**9.0E-07 ± 2.5E-08**	**16.0 ± 0.7**	**5.6E-08 ± 2.8E-09**	4.9
		11-12	**4.1E-06 ± 1.0E-07**	**97.5 ± 2.68**	**4.2E-08 ± 1.6E-09**	3.8
		12-13	**1.5E-06 ± 3.8E-08**	**28.4 ± 1.0**	**5.4E-08 ± 2.3E-09**	4.2
		13-14	**9.3E-07 ± 2.7E-08**	**17.1 ± 0.7**	**5.4E-08 ± 2.7E-09**	4.9
		14-15	**5.1E-07 ± 1.6E-08**	**8.7 ± 0.4**	**5.8E-08 ± 3.5E-09**	5.9
		15-16	**3.6E-07 ± 1.3E-08**	**7.5 ± 0.4**	**4.8E-08 ± 3.1E-09**	6.5
		16-17	**3.3E-07 ± 1.3E-08**	**7.0 ± 0.4**	**4.7E-08 ± 3.2E-09**	6.8
		17-18	**3.0E-07 ± 1.2E-08**	**5.9 ± 0.4**	**5.1E-08 ± 3.6E-09**	7.1
		18-19	**2.0E-07 ± 9.5E-09**	**2.8 ± 0.2**	**7.2E-08 ± 7.0E-09**	9.6
		19-20	**9.4E-08 ± 4.8E-09**	**1.8 ± 0.2**	**5.3E-08 ± 6.3E-09**	12
		20-21	**9.7E-08 ± 3.9E-09**	**2.4 ± 0.2**	**4.0E-08 ± 4.0E-09**	9.8
		21-22	**1.3E-07 ± 5.4E-09**	**3.1 ± 0.2**	**4.4E-08 ± 3.9E-09**	8.9
		22-23	**8.9E-08 ± 3.3E-09**	**2.9 ± 0.2**	**3.1E-08 ± 2.8E-09**	9.0
		23-24	**6.2E-08 ± 3.0E-09**	**0.78 ± 0.13**	**8.0E-08 ± 1.4E-08**	18
	March 16	01-02	*8.0E-09 ± 5.5E-09*	*0.13 ± 0.06*	*6.4E-08 ± 5.4E-08*	85
		03-04	*1.7E-07 ± 4.4E-09*	*0.63 ± 0.12*	*2.7E-07 ± 5.2E-08*	19
		07-08	*1.0E-07 ± 6.1E-09*	*0.42 ± 0.11*	*2.5E-07 ± 6.6E-08*	27
		09-10	*5.1E-07 ± 1.6E-08*	*0.29 ± 0.09*	*1.8E-06 ± 6.0E-07*	33
		11-12	*1.4E-07 ± 6.8E-09*	*0.26 ± 0.08*	*5.2E-07 ± 1.6E-07*	31
	March 20	13-14	**1.0E-07 ± 4.2E-09**	**2.5 ± 0.2**	**4.1E-08 ± 4.1E-09**	9.9
		14-15	**8.1E-08 ± 2.7E-09**	**1.6 ± 0.2**	**5.1E-08 ± 5.9E-09**	12
		15-16	**1.3E-07 ± 8.7E-09**	**2.5 ± 0.2**	**5.3E-08 ± 5.9E-09**	11
		16-17	**2.0E-07 ± 9.4E-09**	**3.6 ± 0.3**	**5.4E-08 ± 4.8E-09**	8.8
		17-18	**2.6E-07 ± 1.0E-08**	**6.9 ± 0.4**	**3.7E-08 ± 2.6E-09**	6.9
		18-19	**2.2E-07 ± 1.0E-08**	**5.4 ± 0.3**	**4.1E-08 ± 3.2E-09**	7.7
		19-20	**2.2E-07 ± 1.1E-08**	**5.6 ± 0.3**	**4.0E-08 ± 3.1E-09**	7.9
		20-21	**2.1E-07 ± 1.0E-08**	**4.9 ± 0.3**	**4.3E-08 ± 3.4E-09**	8.0
		21-22	**1.9E-07 ± 8.8E-09**	**4.6 ± 0.3**	**4.1E-08 ± 3.3E-09**	8.1
		22-23	**2.2E-07 ± 1.1E-08**	**4.5 ± 0.3**	**4.9E-08 ± 4.1E-09**	8.5
		23-24	**1.8E-07 ± 1.0E-08**	**3.6 ± 0.3**	**5.0E-08 ± 4.8E-09**	9.5
		00-01	**2.0E-07 ± 1.1E-08**	**4.1 ± 0.3**	**4.8E-08 ± 4.2E-09**	8.9
		01-02	**3.8E-07 ± 1.4E-08**	**5.5 ± 0.3**	**6.8E-08 ± 4.9E-09**	7.2
		02-03	**3.0E-07 ± 1.2E-08**	**5.5 ± 0.3**	**5.5E-08 ± 4.1E-09**	7.4
		03-04	**4.9E-07 ± 1.6E-08**	**10.0 ± 0.5**	**4.9E-08 ± 2.9E-09**	5.9
		04-05	**5.3E-07 ± 2.0E-08**	**12.8 ± 0.6**	**4.1E-08 ± 2.4E-09**	5.8
		05-06	**4.1E-07 ± 1.6E-08**	**8.5 ± 0.4**	**4.9E-08 ± 3.1E-09**	6.3
		06-07	**3.2E-07 ± 1.3E-08**	**7.2 ± 0.4**	**4.5E-08 ± 3.0E-09**	6.8
		07-08	**2.8E-07 ± 1.4E-08**	**5.2 ± 0.3**	**5.4E-08 ± 4.3E-09**	7.9
		08-09	**1.6E-07 ± 9.8E-09**	**3.7 ± 0.3**	**4.3E-08 ± 4.1E-09**	9.6
		09-10	**8.5E-08 ± 8.3E-09**	**2.0 ± 0.2**	**4.3E-08 ± 6.1E-09**	14
						
07-043	March 12	20-21	**5.8E-07 ± 1.8E-08**	**2.1 ± 0.1**	**2.8E-07 ± 1.8E-08**	6.7
		21-22	**1.5E-06 ± 6.2E-08**	**7.8 ± 0.2**	**2.0E-07 ± 1.0E-08**	5.1
		22-23	**1.2E-05 ± 1.8E-07**	**78.1 ± 1.5**	**1.5E-07 ± 3.8E-09**	2.5
		23-24	**2.6E-05 ± 7.3E-07**	**157 ± 3**	**1.7E-07 ± 5.6E-09**	3.4
	March 13	00-01	**2.1E-05 ± 3.0E-07**	**138 ± 2**	**1.5E-07 ± 3.5E-09**	2.3
		01-02	**6.3E-06 ± 1.2E-07**	**44.0 ± 0.8**	**1.4E-07 ± 3.9E-09**	2.7
		02-03	**1.7E-06 ± 5.2E-08**	**13.8 ± 0.4**	**1.2E-07 ± 5.0E-09**	4.0
		03-04	**6.4E-07 ± 2.7E-08**	**4.1 ± 0.2**	**1.6E-07 ± 9.3E-09**	6.0
	March 14	10-11	**8.5E-07 ± 2.5E-08**	**1.0 ± 0.1**	**8.5E-07 ± 7.4E-08**	8.7
		11-12	*2.6E-07 ± 9.0E-09*	*< 0.09*		
	March 18	16-17	*1.1E-07 ± 5.2E-09*	*0.23 ± 0.04*	*4.8E-07 ± 7.7E-08*	16
		17-18	*3.6E-07 ± 1.1E-08*	*0.68 ± 0.05*	*5.3E-07 ± 3.9E-08*	7.4
		18-19	**8.8E-07 ± 2.3E-08**	**5.7 ± 0.2**	**1.5E-07 ± 6.3E-09**	4.1
		19-20	*5.5E-07 ± 1.5E-08*	*2.4 ± 0.1*	*2.3E-07 ± 1.0E-08*	4.5
		20-21	*3.6E-07 ± 1.0E-08*	*0.55 ± 0.05*	*6.5E-07 ± 6.4E-08*	9.8
		21-22	*9.6E-08 ± 4.0E-09*	*0.24 ± 0.03*	*4.0E-07 ± 5.5E-08*	14
	March 19	08-09	*6.9E-08 ± 3.9E-09*	*< 0.06*		
		09-10	**3.8E-07 ± 1.2E-08**	**3.2 ± 0.1**	**1.2E-07 ± 5.4E-09**	4.5
		10-11	**1.7E-06 ± 4.1E-08**	**21.3 ± 0.5**	**7.7E-08 ± 2.7E-09**	3.5
		11-12	**6.8E-06 ± 1.8E-07**	**108 ± 2**	**6.3E-08 ± 2.1E-09**	3.3
		12-13	**3.1E-06 ± 7.3E-08**	**55.3 ± 1.1**	**5.5E-08 ± 1.7E-09**	3.2
		13-14	*8.3E-08 ± 4.0E-09*	*0.2 ± 0.1*	*3.5E-07 ± 7.6E-08*	22
		14-15	*7.0E-08 ± 4.2E-09*	*< 0.08*		
	March 20	17-18	*1.2E-07 ± 5.4E-09*	*0.52 ± 0.11*	*2.2E-07 ± 4.7E-08*	21
		18-19	**7.8E-07 ± 1.7E-08**	**28.0 ± 1.0**	**2.8E-08 ± 1.1E-09**	4.1
		19-20	**3.3E-06 ± 6.1E-08**	**108 ± 3**	**3.1E-08 ± 1.0E-09**	3.3
		20-21	**1.6E-06 ± 2.9E-08**	**49.9 ± 1.5**	**3.1E-08 ± 1.1E-09**	3.6
		21-22	**4.8E-07 ± 1.1E-08**	**10.3 ± 0.5**	**4.7E-08 ± 2.5E-09**	5.3
		22-23	**9.8E-08 ± 5.3E-09**	**1.0 ± 0.1**	**9.9E-08 ± 1.6E-08**	16
		23-24	**8.8E-08 ± 5.0E-09**	**0.66 ± 0.12**	**1.3E-07 ± 2.6E-08**	19
	March 21	00-01	**8.7E-08 ± 5.2E-09**	**1.5 ± 0.2**	**5.7E-08 ± 7.4E-09**	13
		01-02	**1.2E-07 ± 6.4E-09**	**3.8 ± 0.3**	**3.3E-08 ± 2.9E-09**	8.9
		02-03	**9.8E-08 ± 5.2E-09**	**2.6 ± 0.2**	**3.8E-08 ± 3.9E-09**	10
		03-04	**8.0E-08 ± 4.0E-09**	**2.4 ± 0.2**	**3.3E-08 ± 3.5E-09**	10
		04-05	**1.3E-07 ± 6.1E-09**	**1.3 ± 0.2**	**9.9E-08 ± 1.3E-08**	13
		05-06	*1.0E-07 ± 6.0E-09*	*0.78 ± 0.13*	*1.3E-07 ± 2.2E-08*	17
		06-07	**1.8E-07 ± 6.7E-09**	**1.1 ± 0.1**	**1.7E-07 ± 2.4E-08**	14
		07-08	**1.7E-07 ± 5.8E-09**	**1.5 ± 0.2**	**1.1E-07 ± 1.3E-08**	12
		08-09	**3.3E-07 ± 8.1E-09**	**6.1 ± 0.4**	**5.4E-08 ± 3.4E-09**	6.4
		09-10	**4.8E-07 ± 1.2E-08**	**10.7 ± 0.5**	**4.5E-08 ± 2.4E-09**	5.4
		10-11	**6.7E-07 ± 1.6E-08**	**10.1 ± 0.5**	**6.6E-08 ± 3.5E-09**	5.3
		11-12	**6.3E-07 ± 1.8E-08**	**7.0 ± 0.4**	**9.1E-08 ± 5.7E-09**	6.2
		12-13	**2.6E-07 ± 7.8E-09**	**1.0 ± 0.1**	**2.4E-07 ± 3.6E-08**	15
		13-14	*1.3E-07 ± 5.3E-09*	*1.7 ± 0.2*	*7.5E-08 ± 8.8E-09*	12
		14-15	*7.1E-08 ± 5.0E-09*	*0.53 ± 0.12*	*1.3E-07 ± 3.3E-08*	24
08-009	March 15	08-09	**1.2E-05 ± 2.0E-07**	**153 ± 5**	**7.9E-08 ± 2.8E-09**	3.5
	March 21	04-05	*1.6E-07 ± 5.8E-09*	*1.2 ± 0.2*	*1.4E-07 ± 2.0E-08*	14
		05-06	*4.5E-07 ± 1.3E-08*	*2.7 ± 0.2*	*1.7E-07 ± 1.5E-08*	9.0
		06-07	**1.1E-05 ± 1.7E-07**	**127 ± 3**	**8.9E-08 ± 2.4E-09**	2.7
		07-08	**2.2E-05 ± 2.4E-07**	**309 ± 6**	**7.2E-08 ± 1.7E-09**	2.3
		08-09	**1.4E-05 ± 2.2E-07**	**153 ± 3**	**9.5E-08 ± 2.5E-09**	2.6
		09-10	*2.7E-06 ± 4.7E-08*	*16.0 ± 0.6*	*1.7E-07 ± 6.8E-09*	4.1
		13-14	**3.9E-07 ± 9.9E-09**	**4.3 ± 0.3**	**9.0E-08 ± 6.3E-09**	7.0
		14-15	**3.0E-07 ± 7.8E-09**	**3.4 ± 0.3**	**8.8E-08 ± 6.8E-09**	7.8
		15-16	*2.1E-07 ± 6.9E-09*	*1.1 ± 0.2*	*2.0E-07 ± 3.0E-08*	15
		16-17	*3.1E-07 ± 8.0E-09*	*1.6 ± 0.2*	*1.9E-07 ± 2.2E-08*	12
08-010	March 15	08-09	**6.5E-06 ± 1.2E-07**	**141 ± 3**	**4.6E-08 ± 1.3E-09**	2.8
	March 21	07-08	**1.1E-05 ± 1.9E-07**	**285 ± 6**	**3.7E-08 ± 1.0E-09**	2.7
						
08-011	March 15	10-11	**6.0E-06 ± 4.6E-07**	**76.0 ± 2.2**	**7.8E-08 ± 6.5E-09**	8.2
08-015	March 15	09-10	**4.6E-06 ± 8.9E-08**	**76.6 ± 1.8**	**6.1E-08 ± 1.9E-09**	3.1
08-022	March 15	08-09	**3.5E-06 ± 5.6E-08**	**56 ± 1**	**6.2E-08 ± 1.9E-09**	3.0
	March 21	04-05	*3.3E-07 ± 7.5E-09*	*1.5 ± 0.2*	*2.2E-07 ± 2.6E-08*	12
		05-06	*1.2E-06 ± 2.2E-08*	*2.0 ± 0.2*	*6.3E-07 ± 6.4E-08*	10
		07-08	**8.5E-06 ± 1.3E-07**	**158 ± 3**	**5.4E-08 ± 1.4E-09**	2.6
		08-09	**1.4E-05 ± 1.6E-07**	**244 ± 5**	**5.9E-08 ± 1.4E-09**	2.3
		09-10	**6.0E-06 ± 4.7E-08**	**66.7 ± 1.6**	**9.1E-08 ± 2.3E-09**	2.6
		10-11	**8.6E-07 ± 8.1E-09**	**5.8 ± 0.3**	**1.5E-07 ± 8.5E-09**	5.7
08-023	March 20	13-14	**1.8E-06 ± 1.9E-07**	**21.7 ± 0.7**	**8.19E-08 ± 9.1E-09**	11
08-041	March 15	08-09	**2.6E-06 ± 2.0E-07**	**31.5 ± 1.1**	**8.1E-08 ± 6.9E-09**	8.5
	March 21	07-08	**2.1E-06 ± 3.5E-08**	**38.9 ± 1.3**	**5.3E-08 ± 2.0E-09**	3.7
11-057	March 22	14-15	*1.2E-07 ± 3.4E-09*	*< 0.1*		
		15-16	*1.8E-07 ± 4.6E-09*	*< 0.09*		
		22-23	**4.7E-07 ± 7.2E-09**	**0.64 ± 0.06**	**7.3E-07 ± 6.6E-08**	9.1
		23-14	**3.4E-07 ± 5.4E-09**	**0.34 ± 0.04**	**1.0E-06 ± 1.3E-07**	13
						
11-064	March15	08-09	**3.3E-07 ± 1.4E-08**	**8.3 ± 0.5**	**4.0E-08 ± 2.8E-09**	7.0
		09-10	**2.9E-06 ± 5.8E-08**	**71.3 ± 2.0**	**4.0E-08 ± 1.4E-09**	3.5
		10-11	**1.7E-07 ± 8.8E-09**	**4.4 ± 0.3**	**3.8E-08 ± 3.5E-09**	9.2
	March 20	14-15	**8.1E-07 ± 1.7E-08**	**14.4 ± 0.6**	**5.6E-08 ± 2.8E-09**	4.9
		15-16	**6.7E-07 ± 1.4E-08**	**20.3 ± 0.8**	**3.3E-08 ± 1.5E-09**	4.5
	March 21	05-06	**1.6E-07 ± 2.6E-09**	**2.1 ± 0.2**	**7.5E-08 ± 8.6E-09**	12
		06-07	**1.8E-07 ± 2.8E-09**	**2.6 ± 0.3**	**7.0E-08 ± 7.2E-09**	10
		07-08	**1.9E-07 ± 9.6E-09**	**3.3 ± 0.3**	**5.8E-08 ± 5.8E-09**	10
		08-09	**1.9E-06 ± 3.9E-08**	**70.9 ± 2.0**	**2.7E-08 ± 9.5E-10**	3.5
		09-10	**2.4E-06 ± 4.7E-08**	**88.9 ± 2.5**	**2.7E-08 ± 9.1E-10**	3.4
		10-11	**9.0E-07 ± 3.7E-08**	**30.5 ± 1.1**	**3.0E-08 ± 1.6E-09**	5.4
		11-12	**3.0E-07 ± 1.3E-08**	**4.9 ± 0.3**	**6.1E-08 ± 5.1E-09**	8.5
		12-13	*1.36E-07 ± 7.25E-09*	*1.1 ± 0.2*	*1.2E-07 ± 2.2E-08*	18
12-004	March 16	06-07	*1.2E-08 ± 1.6E-09*	*0.16 ± 0.04*	*7.8E-08 ± 2.4E-08*	31
		07-08	**1.0E-07 ± 3.0E-09**	**0.70 ± 0.08**	**1.5E-07 ± 1.8E-08**	12
		08-09	**3.4E-07 ± 7.6E-09**	**2.2 ± 0.1**	**1.6E-07 ± 9.1E-09**	5.7
		09-10	**8.3E-07 ± 1.6E-08**	**7.0 ± 0.3**	**1.2E-07 ± 4.9E-09**	4.1
		10-11	**3.6E-07 ± 8.1E-09**	**2.3 ± 0.1**	**1.6E-07 ± 8.5E-09**	5.4
		11-12	**1.3E-07 ± 3.8E-09**	**0.99 ± 0.10**	**1.3E-07 ± 1.4E-08**	10
	March 21	06-07	*2.3E-08 ± 1.5E-09*	*0.61 ± 0.12*	*3.7E-08 ± 7.6E-09*	20
		07-08	**3.4E-07 ± 4.1E-09**	**11.6 ± 0.5**	**3.0E-08 ± 1.4E-09**	4.7
		08-09	**2.3E-06 ± 2.7E-08**	**140 ± 4**	**1.6E-08 ± 4.7E-10**	2.9
		09-10	**1.2E-07 ± 2.6E-09**	**8.4 ± 0.4**	**1.5E-08 ± 8.3E-10**	5.5
		10-11	**1.2E-07 ± 2.6E-09**	**6.8 ± 0.4**	**1.8E-08 ± 1.1E-09**	6.0
		11-12	**4.9E-08 ± 2.1E-09**	**1.0 ± 0.2**	**4.7E-08 ± 7.0E-09**	15
12-061	March 16	07-08	**5.5E-07 ± 1.7E-08**	**4.1 ± 0.1**	**1.4E-07 ± 6.4E-09**	4.7
		08-09	**1.3E-06 ± 4.0E-08**	**15.3 ± 0.3**	**8.3E-08 ± 3.0E-09**	3.6
		09-10	**6.3E-07 ± 2.1E-08**	**8.2 ± 0.2**	**7.7E-08 ± 3.1E-09**	4.1
		10-11	**6.2E-07 ± 2.0E-08**	**10.4 ± 0.2**	**6.0E-08 ± 2.3E-09**	3.9
	March 21	06-07	*1.7E-07 ± 2.8E-09*	*1.7 ± 0.1*	*9.9E-08 ± 5.7E-09*	5.8
		07-08	**1.6E-06 ± 4.9E-08**	**78.6 ± 0.6**	**2.0E-08 ± 6.5E-10**	3.2
		08-09	**3.5E-07 ± 1.1E-08**	**11.9 ± 0.2**	**3.0E-08 ± 1.1E-09**	3.8
		09-10	**2.5E-07 ± 8.5E-09**	**12.1 ± 0.2**	**2.0E-08 ± 8.1E-10**	4.0
		10-11	**8.7E-08 ± 2.0E-09**	**2.2 ± 0.1**	**4.0E-08 ± 2.2E-09**	5.4
12-070	March 15	09-10	**2.0E-06 ± 1.8E-07**	**49.2 ± 1.5**	**4.0E-08 ± 3.8E-09**	9.6
	March 21	06-07	**1.2E-07 ± 2.3E-09**	**2.2 ± 0.2**	**5.2E-08 ± 5.9E-09**	11
		07-08	**6.0E-07 ± 6.3E-09**	**18.6 ± 0.8**	**3.2E-08 ± 1.3E-09**	4.2
		08-09	**4.2E-06 ± 3.9E-08**	**161 ± 4**	**2.6E-08 ± 7.1E-10**	2.7
		09-10	**2.8E-06 ± 2.8E-08**	**105 ± 3**	**2.6E-08 ± 7.6E-10**	2.9
		10-11	**6.0E-07 ± 6.3E-09**	**15.8 ± 0.7**	**3.8E-08 ± 1.7E-09**	4.4
		11-12	**1.6E-07 ± 2.7E-09**	**1.8 ± 0.2**	**8.6E-08 ± 1.1E-08**	13
12-084	March 21	08-09	**9.1E-08 ± 2.9E-09**	**1.4 ± 0.2**	**6.6E-08 ± 9.1E-09**	14
		09-10	**6.1E-07 ± 6.7E-09**	**26.0 ± 0.8**	**2.3E-08 ± 7.4E-10**	3.2
		10-11	**2.0E-07 ± 3.5E-09**	**5.6 ± 0.4**	**3.6E-08 ± 2.4E-09**	6.6
		11-12	**1.7E-07 ± 3.0E-09**	**1.3 ± 0.2**	**1.4E-07 ± 1.9E-08**	14
12-093	March 20	13-14	**2.7E-07 ± 7.6E-09**	**3.8 ± 0.3**	**7.2E-08 ± 6.0E-09**	8.4
		14-15	**1.5E-06 ± 3.2E-08**	**26.0 ± 1.0**	**6.0E-08 ± 2.5E-09**	4.3
		15-16	**1.4E-06 ± 2.9E-08**	**11.6 ± 0.6**	**1.2E-07 ± 6.2E-09**	5.3
13-025	March 15	05-06	**1.0E-06 ± 2.5E-08**	**7.7 ± 0.4**	**1.3E-07 ± 7.5E-09**	5.6
		09-10	**1.2E-06 ± 2.8E-08**	**12.2 ± 0.5**	**9.8E-08 ± 4.3E-09**	4.4
		10-11	**5.0E-06 ± 1.1E-07**	**55.6 ± 1.8**	**8.9E-08 ± 3.5E-09**	3.9
		11-12	**1.1E-06 ± 2.6E-08**	**15.2 ± 0.6**	**7.4E-08 ± 3.4E-09**	4.6
	March 16	04-05	**4.7E-07 ± 1.2E-08**	**4.3 ± 0.2**	**1.1E-07 ± 6.1E-09**	5.5
	March 21	04-05	**2.4E-07 ± 6.8E-09**	**3.6 ± 0.2**	**6.7E-08 ± 3.4E-09**	5.1
		05-06	**2.7E-07 ± 7.5E-09**	**4.0 ± 0.2**	**6.6E-08 ± 3.4E-09**	5.1
		06-07	**2.8E-07 ± 8.1E-09**	**3.3 ± 0.1**	**8.4E-08 ± 4.1E-09**	4.9
		07-08	**2.8E-07 ± 8.5E-09**	**3.5 ± 0.2**	**8.1E-08 ± 4.3E-09**	5.3
		08-09	**3.4E-07 ± 9.3E-09**	**4.1 ± 0.1**	**8.2E-08 ± 3.6E-09**	4.4
		09-10	**8.3E-07 ± 2.1E-08**	**8.7 ± 0.3**	**9.5E-08 ± 4.0E-09**	4.2
		10-11	**3.4E-07 ± 9.7E-09**	**5.2 ± 0.2**	**6.5E-08 ± 2.9E-09**	4.4
		11-12	**6.3E-07 ± 1.7E-08**	**6.8 ± 0.2**	**9.3E-08 ± 3.8E-09**	4.1
		12-13	**4.5E-07 ± 1.2E-08**	**3.8 ± 0.2**	**1.2E-07 ± 5.8E-09**	4.9

a Values in bold are judged to be free from cross-contamination and used in the discussion, while those in italics are suspected to be cross-contaminated and should be regarded as inaccurate values. A number of aEb denotes ax10^b^. Note that the data may be revised after re-checking in the future.

b Corresponding to the numbers shown in Fig. 1 of Ebihara et al. (2022) [1].

c "A-B" stands for the collecting time of SPM from A to B, where A and B are hours in 24-hour notation.

d Errors due to counting statistics (1s) in AMS. "E-a" means 10^-a^.

e Errors due to counting statistics (1s) in gamma-ray spectrometry.

f Propagated errors (1s).

g Relative errors in %.

**Table 3:** Hourly atmospheric concentrations of ^129^I and ^137^Cs and their activity ratios for SPM samples collected with PTFE filter tapes^a^.

					^129^I/^137^Cs	

Sitecode ID #^b^	Sampling Date	Sampling time^c^	^129^I (Bq/m^3^)^d^	^137^Cs (Bq/m^3^)^e^	(Bq/Bq)^f^	RE (%)^g^
07-002	March 15	18-19	***7.1E-07 ± 1.6E-08***	**5.4 ± 0.3**	***1.3E-07 ± 7.0E-09***	5.3
		19-20	***1.2E-06 ± 2.5E-08***	**9.3 ± 0.3**	***1.3E-07 ± 5.6E-09***	4.2
		20-21	***1.2E-06 ± 2.2E-08***	**17 ± 1**	***6.6E-08 ± 2.5E-09***	3.7
		21-22	***9.6E-07 ± 1.7E-08***	**33 ± 1**	***2.9E-08 ± 1.1E-09***	3.8
		22-23	***4.5E-07 ± 1.1E-08***	**8.7 ± 0.4**	***5.2E-08 ± 2.6E-09***	5.0
	March 20	13-14	*1.1E-07 ± 4.5E-09*	*1.0 ± 0.1*	*1.2E-07 ± 1.1E-08*	9.6
		14-15	***4.3E-06 ± 1.3E-07***	**44.9 ± 1.4**	***9.5E-08 ± 4.1E-09***	4.3
		15-16	***8.7E-07 ± 1.7E-08***	**35.0 ± 1.2**	***2.5E-08 ± 1.0E-09***	4.0
		17-18	***3.7E-07 ± 9.0E-09***	**12.5 ± 0.5**	***3.0E-08 ± 1.4E-09***	4.7
		20-21	***4.2E-07 ± 9.1E-09***	**19.8 ± 0.7**	***2.1E-08 ± 8.9E-10***	4.2
		23-24	***3.7E-07 ± 8.2E-09***	**19.1 ± 1.2**	***1.9E-08 ± 1.3E-09***	6.9
	March 21	02-03	***6.0E-07 ± 1.2E-08***	**27.8 ± 0.9**	***2.2E-08 ± 8.5E-10***	4.0
		05-06	***8.4E-07 ± 1.6E-08***	**32.2 ± 1.1**	***2.6E-08 ± 1.0E-09***	3.9
07-034	March 12	20-21	*3.8E-07 ± 1.3E-08*	*1.6 ± 0.1*	*2.4E-07 ± 1.4E-08*	5.7
		21-22	*4.2E-06 ± 8.7E-08*	*29.8 ± 0.6*	*1.4E-07 ± 3.9E-09*	2.8
		22-23	***2.5E-05 ± 3.7E-07***	**180 ± 3**	***1.4E-07 ± 3.3E-09***	2.4
		23-24	***1.8E-05 ± 3.7E-07***	**183 ± 3**	***1.0E-07 ± 2.8E-09***	2.8
	March 13	00-01	***1.9E-05 ± 2.8E-07***	**176 ± 3**	***1.1E-07 ± 2.5E-09***	2.4
		01-02	***1.3E-05 ± 2.4E-07***	**109 ± 2**	***1.1E-07 ± 3.1E-09***	2.7
		02-03	***3.8E-06 ± 1.1E-07***	**31.3 ± 0.6**	***1.2E-07 ± 4.4E-09***	3.6
		03-04	***6.7E-07 ± 3.2E-08***	**7.6 ± 0.3**	***8.8E-08 ± 5.2E-09***	5.9
		04-05	***1.7E-06 ± 5.2E-08***	**12.1 ± 0.3**	***1.4E-07 ± 5.8E-09***	4.1
		05-06	***7.5E-07 ± 3.2E-08***	**6.0 ± 0.2**	***1.2E-07 ± 6.6E-09***	5.3
		06-07	***1.6E-06 ± 6.7E-08***	**7.0 ± 0.2**	***2.3E-07 ± 1.2E-08***	5.2
	March 14	09-10	***1.8E-06 ± 2.1E-08***	**9.1 ± 0.3**	***2.0E-07 ± 6.6E-09***	3.3
		10-11	***4.4E-06 ± 5.0E-08***	**18.7 ± 0.4**	***2.3E-07 ± 6.2E-09***	2.7
		11-12	***1.6E-06 ± 2.5E-08***	**7.5 ± 0.2**	***2.1E-07 ± 7.4E-09***	3.6
		12-13	*1.1E-07 ± 3.1E-09*	*0.19 ± 0.04*	*5.9E-07 ± 1.3E-07*	21
	March 18	08-09	*7.1E-08 ± 2.6E-09*	*0.58 ± 0.06*	*1.2E-07 ± 1.3E-08*	11
		09-10	*1.2E-07 ± 3.1E-09*	*0.39 ± 0.06*	*3.1E-07 ± 4.6E-08*	15
		10-11	*6.7E-08 ± 2.4E-09*	*0.36 ± 0.06*	*1.9E-07 ± 3.1E-08*	17
		11-12	*6.6E-08 ± 2.8E-09*	*0.60 ± 0.07*	*1.1E-07 ± 1.3E-08*	12
		16-17	*6.7E-08 ± 2.7E-09*	*0.56 ± 0.07*	*1.2E-07 ± 1.5E-08*	12
		17-18	***4.3E-06 ± 1.2E-07***	**158 ± 3**	***2.7E-08 ± 9.2E-10***	3.4
		18-19	***5.7E-06 ± 1.5E-07***	**148 ± 3**	***3.9E-08 ± 1.3E-09***	3.3
		19-20	***1.8E-06 ± 2.6E-08***	**49.1 ± 1.0**	***3.7E-08 ± 9.3E-10***	2.5
		20-21	***2.9E-07 ± 5.4E-09***	**10.2 ± 0.3**	***2.8E-08 ± 9.9E-10***	3.5
		21-22	*1.9E-07 ± 8.0E-09*	*5.5 ± 0.2*	3.4E-08 ± 1.9E-09	5.7
	March 19	09-10	*3.7E-07 ± 6.0E-09*	*5.4 ± 0.2*	6.9E-08 ± 2.8E-09	4.0
		10-11	***2.2E-06 ± 1.8E-08***	**33.6 ± 0.7**	***6.4E-08 ± 1.5E-09***	2.3
		11-12	***5.7E-06 ± 1.5E-07***	**121 ± 2**	***4.7E-08 ± 1.5E-09***	3.3
		12-13	***2.6E-06 ± 4.0E-08***	**50.6 ± 1.0**	***5.2E-08 ± 1.3E-09***	2.4
		13-14	*1.3E-07 ± 3.4E-09*	*0.71 ± 0.07*	*1.8E-07 ± 1.9E-08*	10
		14-15	*2.0E-07 ± 4.9E-09*	*2.3 ± 0.1*	*8.6E-08 ± 5.2E-09*	6.0
		16-17	*1.7E-07 ± 4.1E-09*	*1.8 ± 0.1*	*9.5E-08 ± 6.5E-09*	6.9
		17-18	*1.3E-07 ± 3.6E-09*	*1.1 ± 0.1*	*1.2E-07 ± 1.0E-08*	8.3
	March 20	11-12	*1.5E-07 ± 3.8E-09*	*0.93 ± 0.14*	*1.6E-07 ± 2.4E-08*	15
		17-18	***3.4E-06 ± 9.1E-08***	**220 ± 6**	***1.6E-08 ± 5.8E-10***	3.7
		18-19	***8.0E-06 ± 2.1E-07***	**357 ± 9**	***2.2E-08 ± 8.1E-10***	3.6
		19-20	***6.7E-06 ± 1.9E-07***	**360 ± 9**	***1.9E-08 ± 7.0E-10***	3.7
		20-21	***4.2E-06 ± 1.2E-07***	**210 ± 5**	***2.0E-08 ± 7.9E-10***	3.9
		21-22	***1.6E-06 ± 3.1E-08***	**65.0 ± 1.9**	***2.5E-08 ± 8.6E-10***	3.5
		22-23	*3.8E-07 ± 5.1E-09*	*6.3 ± 0.4*	*6.0E-08 ± 3.6E-09*	6.0
		23-24	*2.3E-07 ± 4.2E-09*	*5.3 ± 0.3*	*4.3E-08 ± 2.8E-09*	6.5
	March 21	00-01	*3.8E-07 ± 5.7E-09*	*8.9 ± 0.4*	*4.3E-08 ± 2.2E-09*	5.3
		01-02	***6.8E-07 ± 9.5E-09***	**16.4 ± 0.7**	**4.2E-08 ± 1.8E-09**	4.3
		02-03	*4.0E-07 ± 5.5E-09*	*3.7 ± 0.3*	*1.1E-07 ± 8.2E-09*	7.5
		03-04	*1.8E-07 ± 3.8E-09*	*3.8 ± 0.3*	*4.7E-08 ± 3.6E-09*	7.7
		04-05	***4.9E-07 ± 5.9E-09***	**8.5 ± 0.4**	***5.8E-08 ± 3.0E-09***	5.3
		05-06	*2.8E-07 ± 8.7E-09*	*4.4 ± 0.3*	*6.3E-08 ± 4.8E-09*	7.5
		11-12	*4.6E-07 ± 1.2E-08*	*5.6 ± 0.3*	*8.3E-08 ± 5.6E-09*	6.7
08-032	March 16	08-09	***2.9E-06 ± 6.2E-08***	**175 ± 3**	***1.7E-08 ± 4.7E-10***	2.9
	March 20	12-13	***5.8E-07 ± 2.2E-08***	**29.9 ± 0.6**	***1.9E-08 ± 8.2E-10***	4.2
		13-14	***2.0E-07 ± 5.8E-09***	**10.0 ± 0.3**	***2.0E-08 ± 8.2E-10***	4.2
		14-15	***7.6E-08 ± 3.2E-09***	**5.9 ± 0.2**	***1.3E-08 ± 6.9E-10***	5.3
08-036	March 16	08-09	***5.4E-07 ± 8.1E-08***	**44.2 ± 1.2**	***1.2E-08 ± 1.9E-09***	15
	March 20	11-12	***8.6E-08 ± 3.4E-09***	**7.0 ± 0.4**	***1.2E-08 ± 8.3E-10***	6.7
		12-13	***4.4E-07 ± 3.1E-08***	**37.5 ± 1.1**	***1.2E-08 ± 9.0E-10***	7.6
		13-14	***2.1E-07 ± 6.4E-09***	**14.6 ± 0.6**	***1.5E-08 ± 7.2E-10***	4.9
12-027	March 16	06-07	***3.3E-08 ± 2.3E-09***	**0.91 ± 0.08**	***3.6E-08 ± 4.2E-09***	12
		07-08	***4.0E-07 ± 1.6E-08***	**8.4 ± 0.2**	***4.8E-08 ± 2.3E-09***	4.7
		08-09	***3.7E-07 ± 1.4E-08***	**7.2 ± 0.2**	***5.2E-08 ± 2.4E-09***	4.5
		09-10	***2.5E-06 ± 5.5E-08***	**83.1 ± 0.9**	***3.0E-08 ± 7.3E-10***	2.4
		10-11	***1.6E-06 ± 3.6E-08***	**80.5 ± 0.9**	***2.0E-08 ± 5.0E-10***	2.5
		11-12	***6.0E-07 ± 1.4E-08***	**29.2 ± 0.5**	***2.0E-08 ± 5.8E-10***	2.8
		12-13	***3.2E-07 ± 8.7E-09***	**7.7 ± 0.2**	***4.2E-08 ± 1.7E-09***	4.1
		13-14	*6.9E-08 ± 3.1E-09*	*1.5 ± 0.1*	*4.6E-08 ± 3.0E-09*	6.5
		14-15	*1.4E-08 ± 1.3E-09*	*1.9 ± 0.1*	*7.0E-09 ± 8.0E-10*	12
		16-17	*5.4E-09 ± 1.2E-09*	*0.16 ± 0.04*	*3.3E-08 ± 1.0E-08*	31
12-117	March 20	13-14	***5.9E-07 ± 1.3E-08***	**26.4 ± 0.6**	***2.2E-08 ± 7.0E-10***	3.1
		14-15	***2.3E-07 ± 5.5E-09***	**13.7 ± 0.4**	***1.7E-08 ± 5.9E-10***	3.5
		15-16	***2.3E-07 ± 6.6E-09***	**7.7 ± 0.2**	***3.0E-08 ± 1.2E-09***	4.0
12-118	March 16	06-07	*1.4E-07 ± 5.3E-09*	*2.1 ± 0.1*	*6.8E-08 ± 4.7E-09*	6.8
		07-08	***5.0E-07 ± 1.7E-08***	**12.5 ± 0.3**	***4.0E-08 ± 1.6E-09***	4.1
		08-09	***9.7E-07 ± 3.1E-08***	**31.6 ± 0.7**	***3.1E-08 ± 1.2E-09***	3.9
		09-10	***3.5E-06 ± 1.1E-07***	**182 ± 3**	***1.9E-08 ± 7.0E-10***	3.6
		10-11	***3.2E-07 ± 1.0E-08***	**16.8 ± 0.4**	***1.9E-08 ± 7.9E-10***	4.2
		11-12	***1.0E-07 ± 4.1E-09***	**2.6 ± 0.1**	***3.9E-08 ± 2.4E-09***	6.1
	March 20	12-13	***1.2E-07 ± 3.2E-09***	**5.2 ± 0.2**	***2.3E-08 ± 9.3E-10***	4.1
		13-14	***5.2E-07 ± 1.1E-08***	**23.5 ± 0.5**	***2.2E-08 ± 6.7E-10***	3.0
		14-15	***2.4E-07 ± 5.3E-09***	**9.0 ± 0.2**	***2.6E-08 ± 9.3E-10***	3.5
		15-16	***1.1E-07 ± 4.3E-09***	**7.0 ± 0.2**	***1.5E-08 ± 8.0E-10***	5.3
13-023	March 15	05-06	***3.1E-07 ± 7.3E-09***	**7.4 ± 0.3**	***4.1E-08 ± 1.7E-09***	4.2
		09-10	***2.7E-07 ± 6.1E-09***	**9.7 ± 0.5**	***2.8E-08 ± 1.4E-09***	5.2
		10-11	***1.5E-06 ± 2.8E-08***	**52.3 ± 1.6**	***2.9E-08 ± 1.0E-09***	3.5
		11-12	***3.6E-07 ± 7.8E-09***	**13.5 ± 0.6**	***2.7E-08 ± 1.3E-09***	5.0
	March 16	04-05	*1.2E-07 ± 3.9E-09*	*3.4 ± 0.1*	*3.5E-08 ± 1.9E-09*	5.3
	March 21	04-05	***7.1E-08 ± 3.0E-09***	**2.9 ± 0.2**	***2.4E-08 ± 1.9E-09***	7.7
		05-06	***8.8E-08 ± 3.5E-09***	**4.2 ± 0.3**	***2.1E-08 ± 1.6E-09***	7.7
		06-07	***8.0E-08 ± 3.7E-09***	**3.9 ± 0.2**	***2.0E-08 ± 1.4E-09***	7.0
		07-08	***9.1E-08 ± 3.6E-09***	**4.1 ± 0.2**	***2.2E-08 ± 1.3E-09***	6.0
		08-09	***9.4E-08 ± 4.1E-09***	**4.3 ± 0.2**	***2.2E-08 ± 1.5E-09***	6.9
		09-10	***1.7E-07 ± 4.6E-09***	**8.5 ± 0.3**	***2.0E-08 ± 8.9E-10***	4.6
		10-11	***1.1E-07 ± 4.2E-09***	**5.2 ± 0.2**	***2.1E-08 ± 1.3E-09***	6.1
		11-12	***1.3E-07 ± 4.6E-09***	**6.4 ± 0.3**	***2.0E-08 ± 1.2E-09***	6.1
		12-13	***9.0E-08 ± 3.6E-09***	**3.5 ± 0.2**	***2.6E-08 ± 1.6E-09***	6.1
13-069	March 22	22-23	***1.1E-07 ± 2.3E-09***	**0.27 ± 0.03**	***4.0E-07 ± 5.1E-08***	13
		23-24	***1.1E-07 ± 2.2E-09***	**0.27 ± 0.04**	***4.0E-07 ± 6.0E-08***	15
14-008	March 22	14-15	*5.5E-08 ± 2.6E-09*	< 0.1		
		15-16	*3.2E-08 ± 1.8E-09*	< 0.09		
		22-23	***1.9E-07 ± 3.4E-09***	**0.30 ± 0.04**	***6.3E-07 ± 8.7E-08***	14
		23-24	***2.8E-07 ± 4.6E-09***	**0.46 ± 0.05**	***6.2E-07 ± 6.6E-08***	11

a Values in bold are judged to be free from cross-contamination, while those in italics are suspected to be cross-contaminated and should be regarded as inaccurate values.Values in bold and itarics are regarded as reference values. A number of aEb denotes ax10^b^. Note that the data may be revised after re-checking in the future.

b Corresponding to the numbers shown in Fig. 1 of Ebihara et al. (2022) [1].

c "A-B" stands for the collecting time of SPM from A to B, where A and B are hours in 24-hour notation.

d Errors due to counting statistics (1s) in AMS.

e Errors due to counting statistics (1s) in gamma-ray spectrometry.

f Propagated errors (1s).

g Relative errors in %.

**Table 4a:** Reagent blanks.

					^129^I/Bq/mg I^d^
Run #^a^	I/mg^b^	^129^I/Bq^c^			x 10^−9^
15–02	1.012	2.0 × 10^−9^	±	1.7 × 10^−10^	2.0
	1.016	1.6 × 10^−9^	±	1.5 × 10^−10^	1.5
	11.503	1.6 × 10^−8^	±	1.6 × 10^−9^	1.4
	11.514	1.6 × 10^−8^	±	1.3 × 10^−9^	1.4
15–03	1.107	1.6 × 10^−9^	±	9.0 × 10^−11^	1.4
	1.066	1.4 × 10^−9^	±	8.5 × 10^−11^	1.3
15–04	1.292	1.5 × 10^−9^	±	1.1 × 10^−10^	1.2
16–01	2.350	2.7 × 10^−9^	±	2.0 × 10^−10^	1.2
16–02	2.299	2.6 × 10^−9^	±	2.1 × 10^−10^	1.1
16–03	2.471	3.3 × 10^−9^	±	2.4 × 10^−10^	1.3
16–04	2.550	2.4 × 10^−9^	±	3.5 × 10^−10^	0.94
	22.448	2.4 × 10^−8^	±	1.7 × 10–9	1.1
16–05	2.539	2.4 × 10^−9^	±	1.3 × 10^−10^	0.95
	27.535	3.2 × 10^−8^	±	1.6 × 10^−9^	1.2
16–06	2.556	3.5 × 10^−9^	±	3.0 × 10^−10^	1.4
	27.609	3.4 × 10^−8^	±	2.7 × 10^−9^	1.2
16–07	2.372	3.2 × 10^−9^	±	2.8 × 10^−10^	1.3
	2.272	2.5 × 10^−9^	±	2.5 × 10^−10^	1.1
16–08	2.029	2.7 × 10^−9^	±	3.0 × 10^−10^	1.3
	2.121	2.8 × 10^−9^	±	2.5 × 10^−10^	1.3
16–09	2.148	3.0 × 10^−9^	±	1.6 × 10^−10^	1.4
	11.248	8.5 × 10^−8^	±	5.6 × 10–9	7.6
16–10	2.193	1.3 × 10^−9^	±	7.8 × 10^−11^	0.58
17–01	2.148	2.6 × 10^−9^	±	2.2 × 10^−10^	1.2
	11.248	1.4 × 10^−8^	±	1.1 × 10^−9^	1.3
	2.140	2.9 × 10^−9^	±	2.4 × 10^−10^	1.4
	10.633	1.3 × 10^−8^	±	1.0 × 10^−9^	1.2
	2.137	2.5 × 10^−9^	±	2.3 × 10^−10^	1.2
17–02	2.256	2.4 × 10^−9^	±	1.6 × 10^−10^	1.1
	11.836	1.5 × 10^−8^	±	8.1 × 10^−10^	1.3
17–03	2.200	2.9 × 10^−9^	±	1.9 × 10^−10^	1.3
	10.152	1.5 × 10^−8^	±	9.2 × 10^−9^	1.5
17–04	2.214	3.4 × 10^−9^	±	3.3 × 10^−10^	1.5
	11.011	1.6 × 10^−8^	±	1.6 × 10^−9^	1.5
17–05	2.095	2.4 × 10^−9^	±	1.7 × 10^−10^	1.1
	11.340	1.4 × 10^−8^	±	1.3 × 10^−9^	1.2
17–06	2.160	2.8 × 10^−9^	±	2.6 × 10^−10^	1.3
	11.357	1.4 × 10^−8^	±	9.1 × 10^−10^	1.3
17–07	2.222	2.4 × 10^−9^	±	1.2 × 10^−10^	1.1
17–08	2.161	3.2 × 10^−9^	±	3.6 × 10^−10^	1.5
17–09	2.151	2.5 × 10^−9^	±	2.3 × 10^−10^	1.2
17–10	2.099	2.4 × 10^−9^	±	1.2 × 10^−10^	1.2
17–11	2.179	2.1 × 10^−9^	±	1.9 × 10^−10^	0.98
17–12	2.143	2.7 × 10^−9^	±	1.7 × 10^−10^	1.3
18–01	2.152	2.4 × 10^−9^	±	1.2 × 10^−10^	1.1

a Experimental run ID.

b Mass of stable iodine (^127^I in mg) added as a carrier.

c Propagated uncertainty due to counting statistics for SPM sample and reference sample.

d Uncertainty (not shown) is essentially the same as that for ^129^I activity (in Bq).

**Table 4b:** Procedure blanks including reagent blanks.

					^129^I/Bq/mg I^d^
Run #^a^	I/mg^b^	^129^I/Bq^c^			x 10^−9^
15–02	1.024	1.5 × 10^−9^	±	3.2 × 10^−10^	1.5
15–03	1.165	2.4 × 10^−9^	±	8.8 × 10^−11^	2.0
	1.152	1.9 × 10^−9^	±	1.5 × 10^−10^	1.7
	1.275	2.1 × 10^−9^	±	9.4 × 10^−11^	1.6
	1.280	2.1 × 10^−9^	±	1.2 × 10^−10^	1.7
	1.280	2.1 × 10^−9^	±	1.1 × 10^−10^	1.7
	1.113	1.8 × 10^−9^	±	1.1 × 10^−10^	1.7
15–4	1.280	1.7 × 10^−9^	±	1.2 × 10^−10^	1.3
	1.286	2.0 × 10^−9^	±	1.5 × 10^−10^	1.5
	1.285	2.2 × 10^−9^	±	1.6 × 10^−10^	1.7
16–01	2.402	2.7 × 10^−9^	±	1.9 × 10^−10^	1.1
	2.357	3.0 × 10^−9^	±	2.5 × 10^−10^	1.3
	2.376	3.7 × 10^−9^	±	3.3 × 10^−10^	1.6
16–02	2.283	3.3 × 10^−9^	±	2.1 × 10^−10^	1.4
	2.282	3.6 × 10^−9^	±	2.7 × 10^−10^	1.6
16–03	2.461	2.9 × 10^−9^	±	1.9 × 10^−10^	1.2
	2.453	2.7 × 10^−9^	±	1.7 × 10^−10^	1.1
	2.464	3.0 × 10^−9^	±	2.3 × 10^−10^	1.2
	2.464	2.8 × 10^−9^	±	3.0 × 10^−10^	1.2
16–04	2.552	2.9 × 10^−9^	±	1.9 × 10^−10^	1.1
	2.550	2.8 × 10^−9^	±	3.2 × 10^−10^	1.1
	22.297	3.2 × 10^−8^	±	3.7 × 10^–^^9^	1.4
	22.203	2.8 × 10^−8^	±	3.1 × 10^−9^	1.3
16–05	2.549	2.7 × 10^−9^	±	1.4 × 10^−10^	1.1
	2.547	3.1 × 10^−9^	±	1.6 × 10^−10^	1.2
	27.486	2.5 × 10^−8^	±	1.9 × 10^−9^	0.92
	27.609	3.6 × 10^−8^	±	2.5 × 10^−9^	1.3
16–06	2.548	3.6 × 10^−9^	±	3.1 × 10^−10^	1.4
	2.510	3.6 × 10^−9^	±	3.2 × 10^−10^	1.4
	27.757	3.7 × 10^−8^	±	3.4 × 10^−9^	1.3
	27.646	3.4 × 10^−8^	±	2.2 × 10^−9^	1.2
16–07	2.346	3.7 × 10^−9^	±	3.4 × 10^−10^	1.6
	2.350	3.1 × 10^−9^	±	3.1 × 10^−10^	1.3
	2.348	4.6 × 10^−9^	±	3.7 × 10^−10^	2.0
	2.344	4.1 × 10^−9^	±	3.5 × 10^−10^	1.8
16–08	2.043	3.1 × 10^−9^	±	3.6 × 10^−10^	1.5
	2.053	2.5 × 10^−9^	±	2.7 × 10^−10^	1.2
	11.083	1.5 × 10^−8^	±	1.9 × 10^−9^	1.4
	11.062	1.5 × 10^−8^	±	1.5 × 10^−9^	1.3
16–09	2.157	2.9 × 10^−9^	±	1.4 × 10^−10^	1.4
	2.156	1.8 × 10^−8^	±	1.4 × 10^−9^	8.2
	11.329	1.6 × 10^−8^	±	7.1 × 10^−10^	1.4
	11.313	5.6 × 10^−8^	±	4.8 × 10^−9^	5.0
16–10	2.194	1.5 × 10^−9^	±	8.9 × 10^−11^	0.7
	2.192	1.5 × 10^−9^	±	8.6 × 10^−11^	0.7
17–01	2.157	2.8 × 10^−9^	±	2.5 × 10^−10^	1.3
	2.156	2.6 × 10^−9^	±	2.4 × 10^−10^	1.2
	11.329	1.5 × 10^−8^	±	1.1 × 10^−9^	1.4
	11.313	1.4 × 10^−8^	±	1.0 × 10^−9^	1.2
	2.117	2.2 × 10^−9^	±	2.1 × 10^−10^	1.0
	2.128	2.6 × 10^−9^	±	2.2 × 10^−10^	1.2
17–02	2.310	2.9 × 10^−9^	±	2.0 × 10^−10^	1.3
	11.825	1.5 × 10^−8^	±	8.0 × 10^−10^	1.3
17–03	2.202	4.7 × 10^−9^	±	3.0 × 10^−10^	2.1
	10.103	1.2 × 10^−8^	±	7.8 × 10^−10^	1.2
17–04	2.219	5.4 × 10^−9^	±	4.9 × 10^−10^	2.4
	10.985	1.9 × 10^−9^	±	1.8 × 10^−9^	1.8
17–05	2.122	2.9 × 10^−9^	±	1.3 × 10^−10^	1.4
	2.071	3.3 × 10^−9^	±	2.6 × 10^−10^	1.6
	11.346	1.3 × 10^−8^	±	1.4 × 10^−9^	1.1
	11.323	1.7 × 10^−8^	±	1.5 × 10^−9^	1.5
17–06	2.159	3.2 × 10^−9^	±	1.6 × 10^−10^	1.5
	2.160	3.3 × 10^−9^	±	1.7 × 10^−10^	1.6
	11.351	1.4 × 10^−8^	±	6.1 × 10^−10^	1.2
	11.330	1.4 × 10^−8^	±	1.1 × 10^−9^	1.3
17–07	2.215	2.6 × 10^−9^	±	1.5 × 10^−10^	1.2
	2.223	2.5 × 10^−9^	±	1.4 × 10^−10^	1.1
	2.121	5.3 × 10^−9^	±	6.6 × 10^−10^	2.5
	2.122	2.8 × 10^−9^	±	3.8 × 10^−10^	1.3
17–08	2.162	2.7 × 10^−9^	±	4.0 × 10^−10^	1.3
	2.167	4.7 × 10^−9^	±	5.1 × 10^−10^	2.2
17–09	2.159	2.8 × 10^−9^	±	2.7 × 10^−10^	1.3
	2.159	2.5 × 10^−9^	±	2.7 × 10^−10^	1.2
17–10	2.104	2.5 × 10^−9^	±	2.5 × 10^−10^	1.2
	2.074	3.4 × 10^−9^	±	4.3 × 10^−10^	1.6
17–11	2.174	2.7 × 10^−9^	±	2.4 × 10^−10^	1.2
	2.174	2.7 × 10^−9^	±	2.4 × 10^−10^	1.2
	2.173	3.0 × 10^−9^	±	1.6 × 10^−10^	1.4
	2.171	2.6 × 10^−9^	±	2.3 × 10^−10^	1.2
17–12	2.138	4.4 × 10^−9^	±	5.3 × 10^−9^	2.0
	2.140	3.0 × 10^−9^	±	1.4 × 10^−9^	1.4
18–01	2.180	2.8 × 10^−9^	±	1.5 × 10^−9^	1.3

a Experimental run ID.

b Mass of stable iodine (^127^I in mg) added as a carrier.

c Propagated uncertainty due to counting statistics for SPM sample and reference sample.

d Uncertainty (not shown) is essentially the same as that for ^129^I activity (in Bq).

**Table 4c:** Filter blanks (brand-new filter blanks).

Material^a^	# of filter^b^	size/ mm^c^	I/mg^d^	^129^I/Bq^e^		
For SPM		10 × 10				
GF	1		1.107	1.8 × 10^−9^	±	1.0 × 10^−10^
GF	3		1.118	2.3 × 10^−9^	±	2.5 × 10^−10^
GF	8		1.156	3.0 × 10^−9^	±	6.3 × 10^−10^
PTFE	1		1.168	1.9 × 10^−9^	±	9.8 × 10^−11^
PTFE	3		1.175	1.9 × 10^−9^	±	9.6 × 10^−11^
PTFE	12		1.133	1.9 × 10^−9^	±	1.1 × 10^−10^
For APM^f^		10 Φ				
GF	1		1.184	2.7 × 10^−9^	±	1.9 × 10^−19^
GF	1		1.171	2.0 × 10^−9^	±	1.8 × 10^−10^
GF	1		1.172	2.5 × 10^−9^	±	2.4 × 10^−10^
GF	1		1.172	2.3 × 10^−9^	±	1.9 × 10^−10^
GF	1	(1/2)^g^	1.277	2.1 × 10^−9^	±	2.8 × 10^−10^
GF	1	(1/2)	1.277	3.7 × 10^−9^	±	5.5 × 10^−10^
GF	1	(1/2)	1.28	1.9 × 10^−9^	±	1.4 × 10^−10^
GF	1	(1/2)	1.278	2.2 × 10^−9^	±	2.0 × 10^−10^
GF	1		1.278	3.3 × 10^−9^	±	6.2 × 10^−10^
GF	1		1.278	2.6 × 10^−9^	±	9.3 × 10^−10^
GF	1		1.282	4.0 × 10^−9^	±	5.8 × 10^−10^
GF	1		1.282	3.1 × 10^−9^	±	1.0 × 10^−10^
Qz	1		1.170	2.1 × 10^−9^	±	1.3 × 10^−10^
Qz	1		1.161	2.8 × 10^−9^	±	8.9 × 10^−10^
Qz	1		1.167	2.3 × 10^−9^	±	1.5 × 10^−10^
Qz	1		1.166	4.4 × 10^−9^	±	1.8 × 10^−9^

a Filter material used for collecting SPM and APM. Material for APM was used in Ebihara et al. [2]. Qz: quartz filter.

b The number of filter pieces for a single experimental procedure.

c A square shaped-filter piece was used for SPM, while a circle piece was used for APM.

d Mass of stable iodine (^127^I in mg) added as a carrier.

e Propagated uncertainty due to counting statistics for SPM sample and reference sample.

f Corresponding data were plotted in Fig. 2 of Ebihara et al. [2].

g Half a circle was used for analysis. A ^129^I activity value corresponds to this size.

**Table 4d:** Filter blanks (used-filter blanks (spot blanks)).

SPM	Run					
site #^a^	#^b^	position^c^	I/mg^d^	^129^I/Bq^e^		
GF filter						
04–030	17–04	3/17–18	2.221	2.4 × 10^−8^	±	9.0 × 10^−10^
07–003	17–10	3/14–15	2.112	7.2 × 10^−9^	±	1.8 × 10^−10^
07–004	16–02	3/16–17	2.200	1.9 × 10^−8^	±	7.0 × 10^−10^
07–012	16–02	3/19–20	2.049	1.6 × 10^−8^	±	1.0 × 10^−9^
07–019	16–07	3/23–24	2.373	2.2 × 10^−9^	±	1.2 × 10^−10^
07–020	16–07	3/23–24	2.345	1.2 × 10^−7^	±	3.2 × 10^−9^
07–030	16–08	3/14–15	2.046	6.2 × 10^−9^	±	5.5 × 10^−10^
07–031	16–03	3/21–22	2.461	7.0 × 10^−8^	±	2.5 × 10^−9^
07–032	16–08	3/14–15	2.050	1.0 × 10^−8^	±	6.5 × 10^−10^
07–035	16–08	3/14–15	2.035	5.0 × 10^−7^	±	1.2 × 10^−8^
07–035	16–06	3/23–24	2.515	1.4 × 10^−8^	±	6.7 × 10^−10^
07–037	16–08	3/14–15	2.039	5.0 × 10^−9^	±	4.2 × 10^−10^
07–038	16–08	3/14–15	2.047	6.4 × 10^−9^	±	6.0 × 10^−10^
07–040	16–04	3/23–24	2.546	5.2 × 10^−8^	±	1.8 × 10^−9^
07–042	16–10	3/11–12	2.183	2.0 × 10^−6^	±	7.3 × 10^−8^
07–043	16–04	3/14–15	2.537	1.3 × 10^−8^	±	5.4 × 10^−10^
08–009	16–01	3/20–21	2.339	4.3 × 10^−8^	±	1.2 × 10^−9^
08–009	15–03	3/23–24	1.172	2.6 × 10^−8^	±	3.4 × 10^−10^
08–011	15–03	3/23–24	1.164	2.9 × 10^−8^	±	3.2 × 10^−10^
08–015	15–03	3/23–24	1.170	1.7 × 10^−8^	±	3.4 × 10^−10^
08–022	15–03	3/23–24	1.180	1.0 × 10^−7^	±	1.7 × 10^−9^
08–023	15–03	3/23–24	1.179	8.4 × 10^−9^	±	3.1 × 10^−10^
08–041	16–01	3/29	2.341	2.0 × 10^−8^	±	9.0 × 10^−9^
11–057	15–03	3/23–24	1.173	1.1 × 10^−8^	±	2.4 × 10^−10^
11–064	15–03	3/23–24	1.275	1.1 × 10^−8^	±	3.1 × 10^−10^
12–004	17–10	3/14–15	2.101	3.2 × 10^−9^	±	2.3 × 10^−10^
12–004	15–03	3/23–24	1.275	2.9 × 10^−8^	±	7.3 × 10^−10^
12–061	16–03	3/19–20	2.462	1.8 × 10^−8^	±	7.4 × 10^−10^
12–070	16–01	3/20–21	2.332	1.1 × 10^−8^	±	5.3 × 10^−10^
12–070	15–03	3/23–24	1.277	7.4 × 10^−9^	±	2.7 × 10^−10^
12–084	18–01	3/23–24	2.115	1.9 × 10^−8^	±	5.6 × 10^−10^
12–093	16–02	3/14–15	2.209	1.1 × 10^−8^	±	3.7 × 10^−10^
12–093	15–03	3/23–24	1.278	1.8 × 10^−8^	±	4.0 × 10^−10^
13–025	16–02	3/20–21	2.184	4.7 × 10^−9^	±	2.5 × 10^−10^
13–025	15–03	3/23–24	1.279	1.0 × 10^−8^	±	4.0 × 10^−10^
PTFE filter						
07–002	16–02	3/14–15	2.235	3.6 × 10^−9^	±	1.7 × 10^−10^
07–034	16–04	3/12–13	2.544	8.7 × 10^−9^	±	4.3 × 10^−10^
08–032	15–03	3/14–15	1.159	5.7 × 10^−9^	±	2.5 × 10^−10^
08–036	15–03	3/23–24	1.159	2.7 × 10^−9^	±	1.9 × 10^−10^
12–027	17–09	3/23–24	2.163	3.7 × 10^−9^	±	2.7 × 10^−10^
12–117	15–03	3/14–15	1.276	2.5 × 10^−9^	±	1.2 × 10^−10^
12–117	16–02	3/19–20	2.196	4.2 × 10^−9^	±	2.0 × 10^−10^
12–118	16–02	3/14–15	2.226	3.3 × 10^−9^	±	1.8 × 10^−10^
12–118	15–03	3/23–24	1.278	2.9 × 10^−9^	±	1.2 × 10^−10^
13–023	15–03	3/23–24	1.275	2.7 × 10^−9^	±	1.2 × 10^−10^
13–069	15–03	3/23–24	1.181	2.8 × 10^−9^	±	8.3 × 10^−11^
14–008	15–03	3/23–24	1.174	2.4 × 10^−9^	±	7.3 × 10^−11^

a Site ID of SPM sampling stations (see Table 1).

b Experimental run ID.

c Date corresponding to blank spots. “3/A-B” stands for the space between day A and day B in March.

d Mass of stable iodine (^127^I in mg) added as a carrier.

e Propagated uncertainty due to counting statistics for SPM sample and reference sample.

## 2 Experimental Design, Materials and Methods

### 2.1 Samples

Sampling sites of the SPM samples analyzed for the ^129^I activity are listed in Table 1 and illustrated in Fig. 1of the research article [1]. An SPM sample consists of airborne particulate matter in aerosol (<10 μm) and a filter material (either glass fiber (GF) or polytetrafluoroethylene (PTFE) filter) on which aerosol is accumulated. Each (whole) SPM sample contains aerosol in the atmosphere of about 1 m^3^, accumulated for one hour. The SPM samples analyzed were chosen from those once used for non-destructive determination of the ^137^Cs radioactivity [5,6]. Among them, such samples as those having relatively high ^137^Cs radioactivity concentrations (about 1 Bq/m^3^ and higher) were chosen for the ^129^I activity measurement. A part of the whole circler SPM spot (11 mm; 95 mm^2^) was used for the determination of the ^129^I radioactivity. Usually, a quarter part (∼24 mm^2^ SPM spot on ∼1 x 1 cm filter) was used. For some cases, especially when the ^129^I activity was assumed to be considerably high based on the pre-measured ^137^Cs activity, one eighth was used. Cutting of an SPM sample with filter was done manually with scissors. Prior to the ^129^I determination, each cut piece of the SPM samples was subjected to gamma-ray spectrometry using Ge semiconductor detectors for determining the ^137^Cs activity, followed by radiographical imaging using an imaging plate for identifying the location of beta-ray emitters (mainly ^137^Cs).

### 2.2 Sample Preparation and Processing for AMS

For quantitative determination of the ^129^I in SPM samples by AMS, ^129^I was chemically separated. Chemical separation procedures for ^129^I in SPM were modified from those developed for the determination of trace halogens (chlorine, bromine and iodine) in silicate rock samples including meteorites (e.g., [7,8]). The procedures are essentially the same as those described in [2] and briefly summarized as follows. A cut piece of each SPM filter (a square filter of ∼1 x ∼1 cm with a quadrant of the SPM disk (11 mm for a typical case)) was placed on the bottom of a Ni crucible, in which a known amount KI (∼ 1 or 2 mg of ^127^I) as iodine carrier was taken in solution, alkalized with concentrated NaOH solution and dried on a hot plate, soaked with concentrated NaOH solution and fused with ∼ 1 g of NaOH pellets on a Meker burner. After heating gently for two minutes and, then, intensively for five minutes, the fusion cake formed was disaggregated with water. With centrifugation, the supernatant solution containing ^129^I was separated and neutralized with 6 M HNO_3_ with a certain amount of Na_2_SO_3_ as reductant. By adding AgNO_3_ solution drop by drop to the solution, AgI was precipitated and subjected to AMS. For each analytical run, blank samples of reagents and the whole procedure were prepared and chemically processed along with SPM samples for monitoring the blank contributions of ^129^I. For the reagent blank samples, the same amounts of KI and Na_2_SO_3_ as used for SPM samples were taken. Reagent blank values are summarized in Table 4(a). For the procedure blank samples, the same chemical procedure as applied to SPM samples was performed with the use of chemicals but no filters. Procedure blank values are summarized in Table 4(b). Note that those values include reagent blanks. In addition to reagent and whole procedure blanks, blank contributions from the filter material were monitored. There were two types of filter blanks; ^129^I in brand-new filter and ^129^I in used-filter. For the latter case, an open (marginal) part of the used-filter was sampled with one cut piece per one filter roll. The size of each sample subjected to the AMS analysis was adjusted to that of an SPM sample having a quadrant of each SPM spot (∼1 x 1 cm as a filter size). These blanks were called spot blanks. Filter blanks for bland-new filters and used-filters are given in Table 4(c) and Table 4(d), respectively.

### 2.3 Accelerator Mass Spectrometry (AMS)

The ^129^I activity in each SPM sample was determined based on an isotopic ratio of ^129^I/^127^I determined by AMS and an amount of ^127^I carrier added into a crucible before the alkaline fusion. AMS was performed using a tandem accelerator at the MALT facility of the University of Tokyo [9]. For obtaining reliable values of the ^129^I/^127^I isotopic ratio by the MALT AMS, an isotopic ratio of ^129^I/^127^I is desired to fall between 10^−14^ and 10^−8^. With the use of 1 to 2 mg of ^127^I as a carrier of iodine, most SPM samples analyzed have isotopic ratios of ^129^I/^127^I within this range and, therefore, no further procedure for adjusting the isotopic ratio of iodine was needed.

## Ethics Statements

There are no matters that are ethically problematic.

## CRediT authorship contribution statement

**Mitsuru Ebihara:** Writing – original draft. **Naoki Shirai:** Writing – review & editing. **Yasuji Oura:** Formal analysis, Visualization, Funding acquisition. **Haruo Tsuruta:** Data curation, Visualization. **Hiroyuki Matsuzaki:** Funding acquisition. **Yuichi Moriguchi:** Supervision.

## Declaration of Competing Interest

The authors declare that they have no known competing financial interests or personal relationships which have, or could be perceived to have, influenced the work reported in this article.

## Data Availability

Data of 129I and137Csconcentrations insuspendedparticulate matterdispersed ineastern Japan afterthe 2011 nuclearaccident inFukushima, Japan (Original data) (Mendeley Data).
